# Supramolecular protein assembly supports immobilization of a cytochrome P450 monooxygenase system as water-insoluble gel

**DOI:** 10.1038/srep08648

**Published:** 2015-03-03

**Authors:** Cheau Yuaan Tan, Hidehiko Hirakawa, Teruyuki Nagamune

**Affiliations:** 1 Department of Bioengineering, School of Engineering, The University of Tokyo, 7-3-1 Hongo, Bunkyo-ku, Tokyo 113-8656, Japan; 2 Department of Chemistry and Biotechnology, School of Engineering, The University of Tokyo, 7-3-1 Hongo, Bunkyo-ku, Tokyo 113-8656, Japan

## Abstract

Diverse applications of the versatile bacterial cytochrome P450 enzymes (P450s) are hampered by their requirement for the auxiliary proteins, ferredoxin reductases and ferredoxins, that transfer electrons to P450s. Notably, this limits the use of P450s as immobilized enzymes for industrial purposes. Herein, we demonstrate the immobilization of a bacterial P450 and its redox protein partners by supramolecular complex formation using a self-assembled heterotrimeric protein. Employment of homodimeric phosphite dehydrogenase (PTDH) for cross-linking “proliferating cell nuclear antigen-utilized protein complex of P450 and its two electron transfer-related proteins” (PUPPET) yielded a gelling PUPPET-PTDH system capable of regenerating NADH for electron supply owing to its phosphite oxidation activity. The protein gel catalyzed monooxygenation in the presence of phosphite and NAD^+^. The gel was completely water-insoluble and could be reused. This concept of oligomeric protein-insolubilized enzymes can be widely applied to various multienzymatic reactions such as cascade reactions and coupling reactions.

Enzymes are promising tools for the sustainable manufacture of many chemicals^1^,^2^, notably in the field of fine chemicals^3^,^4^,^5^. Recent advances in enzyme mining and protein engineering now make it easier to obtain industrially-useful enzymes with optimized selectivity, pH and temperature profiles and targeted substrate specificity^6^,^7^. Cytochrome P450 monooxygenases (P450s), which have physiologically important roles ranging from biosynthesis of metabolites^8^ to detoxification of xenobiotics^9^, are attractive enzymes for organic syntheses. Their ability to oxidize a wide range of non-activated compounds with regio/stereo-selectivity makes them valuable to researchers and industrialists alike for utilization in alternative chemical synthetic routes^10^,^11^. Pragmatically, soluble bacterial P450s are of interest in biotechnological applications^12^ because of their high turnover and facile heterologous expression in *Escherichia coli* with ease of handling compared to membrane-bound eukaryotic P450s. Furthermore, directed evolution and mutagenesis studies of bacterial P450s have generated various mutants with improved stability, activity and altered substrate specificity toward unnatural substrates^13^,^14^,^15^.

Immobilization of enzymes is essential for their reuse, long-term operation and separation from products in industries^16^. However, there are only few reports on practical immobilization of bacterial P450s, presumably because of the requirement for auxiliary proteins. Most bacterial P450s form transient protein complexes with electron transfer proteins to accept electrons that are essential for their catalytic cycles. The electron transfer proteins are reduced by their specific reductases and therefore, work as shuttle molecules to carry electrons from the reductases to the P450s^17^. Immobilization approaches that hamper the movement of electron transfer proteins, such as direct cross-linking for co-insolubilization and covalent attachment on a solid support, would prohibit electron transfer from reductases to P450s through electron transfer proteins^17^ though adsorption of cell extracts containing a bacterial P450 on ion exchange resins was reported^18^. Therefore, an unusual bacterial P450, P450 BM3, which is a self-sufficient natural fusion protein of a heme-containing domain and an FAD- and FMN-containing reductase domain^19^ was a major target to be immobilized on supports^20^,^21^,^22^. Nevertheless, co-immobilization of bacterial P450s, their electron transfer proteins and reductases continues to be a great challenge.

In our previous study, a bacterial P450, its electron transfer protein and reductase were genetically fused to a heterotrimeric proliferating cell nuclear antigen (PCNA) from *Sulfolobus solfataricus* to yield the protein complex, PUPPET (PCNA-utilized protein complex of P450 and its two electron transfer-related proteins)^23^ (Fig. 1a). The PCNA is composed of three distinct subunits, PCNA1, PCNA2 and PCNA3, which forms a ring-shaped heterotrimer in a step-wise manner^24^,^25^ (Fig. 1b). The C-termini of all the subunits are exposed on the same side of the ring giving an edge for fusion to a *Pseudomonas putida* P450 (P450cam), its electron transfer protein, putidaredoxin (PdX), and specific reductase, putidaredoxin reductase (PdR), thereby co-localizing the enzymes on the PCNA ring. The electron transfer protein carries electrons from the reductase to the P450 in the complex by shuttling them. Therefore, PUPPET works as a single enzyme, similar to P450 BM3 and P450 RhF^26^ in which the reductase domains and electron transfer domains are naturally fused to the heme domains. The catalytic turnover of PUPPET was much higher (500 min^−1^)^23^ than that of an artificial triple fusion protein of PdR-PdX-P450cam (30 min^−1^)^27^. From this viewpoint, we thought it would be advantageous to utilize PUPPET as a platform for further immobilization studies.

We envisioned that a selective linkage between the PCNA rings should lead to an aggregate of PUPPET without loss of its activity. Though it is difficult to selectively cross-link PCNA subunits in PUPPET with chemical reagents, introduction of more than two assembling domains into the PCNA ring would give rise to a supramolecular formation of PUPPET through a selective linkage between the PCNA subunits. Thinking of a bottom-up approach, the dimerized PCNA subunits are expected to form a massive supramolecule, in which PUPPET can be found at the assembling points. Here, we report a facile approach to immobilize a bacterial P450 system simply by employing the self-assembly nature of two oligomeric proteins. The PCNA subunit proteins genetically fused to a homodimeric protein can spontaneously assemble to form a supramolecular complex (Fig. 1c). Phosphite dehydrogenase (PTDH) from *Pseudomonas stutzeri* was used to obtain homodimeric PCNA subunit proteins because it exists as a stable homodimeric protein and regenerates NADH^28^, which is consumed by P450-catalyzed monooxygenation. The protein mixture resulted in a water-insoluble gel with monooxygenase activity and achieved a cost-effective monooxygenation process as a result of NADH regeneration by PTDH with phosphite^29^,^30^. This is the first report on a bacterial P450 which requires separate redox partners to be immobilized in order to catalyze its monooxygenation reaction with cofactor regeneration. This immobilization method could be widely applied to various multienzymatic reactions such as cascade and coupling reactions because enzymes can be immobilized by fusion to homooligomerized PCNA subunit proteins without physical and chemical contacts.

## Results

### Characterization of PCNA fusion proteins

We previously demonstrated that fusion to the C-termini of cysteine mutants of PCNA1 (PCNA1_G108C_), PCNA2 (PCNA2_L171C_) and PCNA3 (PCNA3_R112C/T180C_) enabled PdR, PdX and P450cam to form a stable heterotrimeric complex^31^. To obtain the homodimerized PCNA subunit proteins, the E175A/A176R mutant of PTDH was genetically linked to the N-termini of PCNA1_G108C_-PdR, PCNA2_L171C_-PdX and PCNA3_R112C/T180C_-P450cam. The three fusion proteins, PTDH-PCNA1_G108C_-PdR, PTDH-PCNA2_L171C_-PdX and PTDH-PCNA3_R112C/T180C_-P450cam, were successfully expressed in *E*. *coli* and purified. The yields of the proteins were 77, 17 and 59 mg for PTDH-PCNA1_G108C_-PdR, PTDH-PCNA2_L171C_-PdX and PTDH-PCNA3_R112C/T180C_-P450cam, respectively in 1 L culture. This yield is in accordance with our previously reported protein PTDH-PCNA2-PdX without cysteine mutant (16 mg/L)^30^. The molecular masses of the fusion proteins under a denatured condition were estimated to be 110, 76 and 120 kDa for PTDH-PCNA1_G108C_-PdR, PTDH-PCNA2_L171C_-PdX and PTDH-PCNA3_R112C/T180C_-P450cam, respectively, by SDS-PAGE analysis (Supplementary Fig. S3), and are in good agreement with the molecular masses calculated from their single polypeptide chains, 109, 75 and 111 kDa. The molecular masses of non-denatured PTDH-PCNA1_G108C_-PdR, PTDH-PCNA2_L171C_-PdX and PTDH-PCNA3_R112C/T180C_-P450cam were estimated to be 480, 400 and 540 kDa, respectively, by size-exclusion chromatography (Supplementary Table 1). The molecular masses of fusion proteins evaluated from size exclusion chromatography were found to significantly deviate from the calculated molecular masses of their homodimers (220 kDa, 154 kDa and 226 kDa, respectively) because these fusion proteins are not globular proteins while the protein standards are globular proteins. Indeed, even though PTDH-PCNA2-PdX fusion protein in which PTDH and PdX were genetically linked to the N-terminus and C-terminus of the wild type PCNA2, respectively, was previously reported to be a homodimer^30^, its molecular weight was estimated to be 370 kDa. The ratio of estimated molecular weights of the three fusion proteins were similar to the ratio calculated from their molecular weights. Therefore, the fusion proteins likely assume a homodimeric state. Furthermore, the homodimeric states of these fusion proteins were also proven by dynamic light scattering measurement (Supplementary Fig. S1). The diameters of PTDH-fused PCNA1_G108C_-PdR, PCNA2_L171C_-PdX and PCNA3_R112C/T180C_-P450cam were double than that of their counterpart without PTDH fusion. These supported our contention of homodimeric states of our triple fusion proteins.

The UV-vis spectra of the three fusion proteins displayed characteristic bands derived from the respective component proteins, PdR, PdX and P450cam (Supplementary Fig. S2). Thus, PdR, PdX and P450cam domains were expected to retain their functions in the fusion proteins. In fact, the ferricyanide reduction activity of the PdR domain in PTDH-PCNA1_G108C_-PdR, the cytochrome c reduction ability of the PdX domain in PTDH-PCNA2_L171C_-PdX and the d-camphor-dependent electron accepting ability of the P450cam domain in PTDH-PCNA3_R112C/T180C_-P450cam were comparable to their native counterparts (Supplementary Table 2). Additionally, the phosphite-dependent NAD^+^ reduction activities of the fusion proteins were found to be similar to that of the PTDH mutant (Supplementary Table 2). These results indicate that all three fusion proteins retained the functions of their counterparts.

### Formation of the immobilized enzyme complex

**Figure 2:**
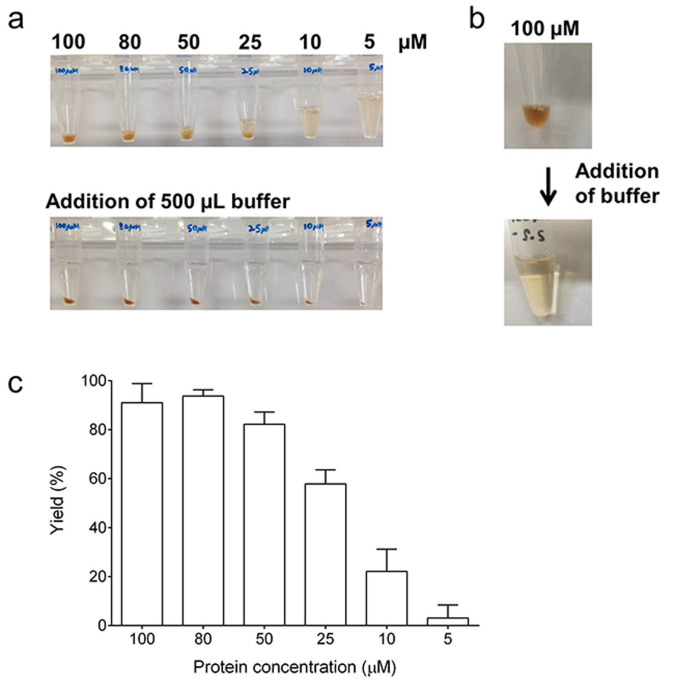
Formation of the water-insoluble gel. (a) Equimolar (100, 80, 50, 25, 10 and 5 μM) mixtures of PTDH-PCNA1_G108C_-PdR, PTDH-PCNA2_L171C_-PdX and PTDH-PCNA3_R112C/T180C_-P450cam were incubated in the presence of 10 mM oxidized glutathione at 4°C overnight (upper panel). The mixtures were centrifuged after the addition of buffer (lower panel). (b) A mixture containing 100 μM PTDH-PCNA1-PdR, 100 μM PTDH-PCNA2-PdX and 100 μM PTDH-PCNA3-P450cam was incubated at 4°C overnight (top) and then centrifuged after addition of buffer (bottom). (c) Yield of gel formation. The yields were determined from the amount of PTDH-PCNA3_R112C/T180C_-P450cam in the combined supernatant and wash.

After overnight incubation with oxidized glutathione at 4°C, equimolar mixtures of PTDH-PCNA1_G108C_-PdR, PTDH-PCNA2_L171C_-PdX and PTDH-PCNA3_R112C/T180C_-P450cam formed a water-insoluble gel (Fig. 2a), which was proven to be comprised of equimolar amounts of the three fusion proteins by analysis of the proteins remaining in the supernatants (Supplementary Fig. S4). In contrast, the incubated mixture of the Cys-free PCNA subunit fusion proteins, PTDH-PCNA1-PdR, PTDH-PCNA2-PdX and PTDH-PCNA3-P450cam, was completely soluble upon addition of buffer even at a high protein concentration of 100 μM (Fig. 2b). The gel formation yield was dependent on the concentration of fusion proteins (Fig. 2c). The mixture containing 100 μM fusion proteins enabled 91% of the fusion proteins to form a gel, while that containing 5 μM fusion proteins yielded a 3% gel. Among the concentrations tested, gel yielded from the mixture containing 80 μM fusion proteins was used for subsequent experiments. The stabilization of the gel was verified with the oxygen consumption activity of the wash solutions of the gel (Supplementary Fig. S5). The activity of the wash solutions of the gel was negligible after 3 rounds of washes indicating that no further leakage of fusion proteins occurred. Thus, the gels were washed 6 rounds prior to any activity measurements.

### Preparation of the small gel

**Figure 3:**
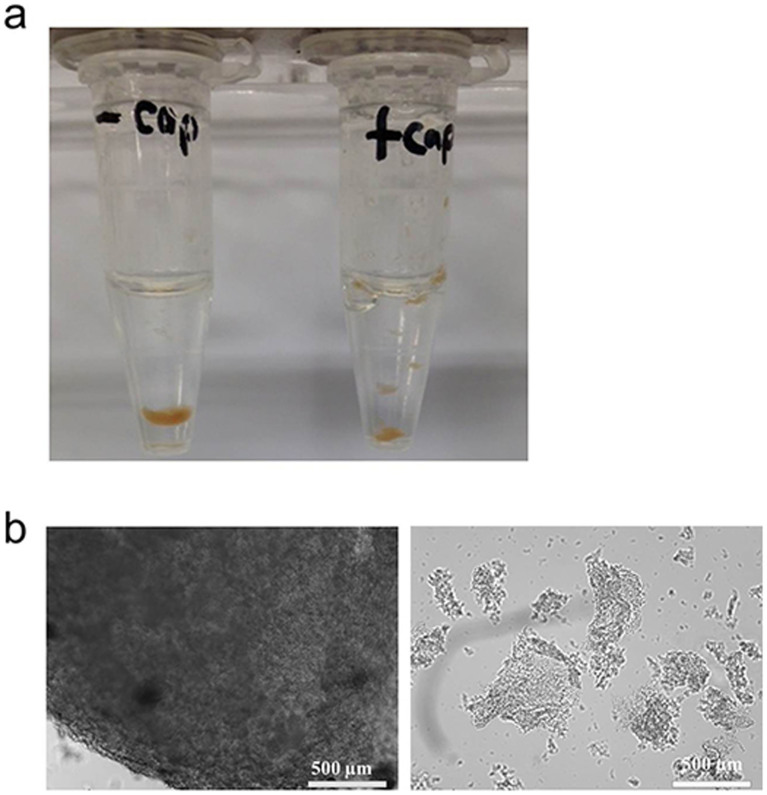
Size difference between the capped and uncapped gels. (a) Picture of uncapped (left) and capped (right) gels after resuspension of the gels with buffer. (b) Microscopic images of uncapped (left) and capped (right) gels. The scale bars indicate 500 μm.

To increase the specific surface area of the gel, we tried to fragmentize it by introducing PCNA2_L171C_ and PCNA3_R112C/T180C_ monomers (“capping molecules”) to the gel after overnight incubation. These capping molecules could potentially bind to the PCNA1 domain and PCNA1-associated PCNA2 domain which could be exposed on the surface of the gel. As predicted, the capping molecules broke the gel into smaller particles and this re-suspension phenomenon was again observed in the second cycle of buffer addition (Fig. 3a). In contrast, the uncapped gel retained its massive form even after similar treatment of rotating the gel in the presence of an equal volume of buffer. Light microscopy observations revealed that the capped gels were small amorphous particles with sizes varying from 400 μm to 800 μm in length and the uncapped gel was a huge piece of mass without distinct gaps (Fig. 3b).

### Activity of the water-insoluble gel

**Figure 4:**
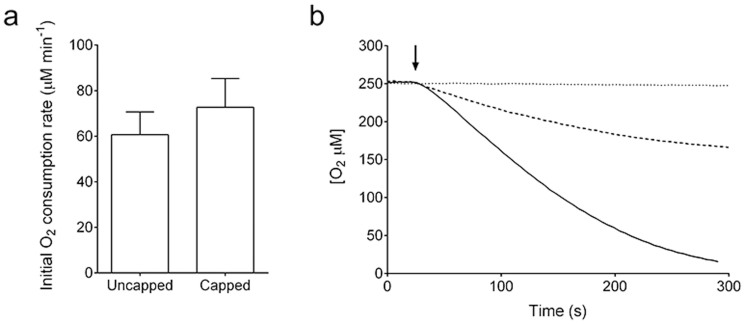
Oxygen consumption activity in the presence of d-camphor. (a) Initial oxygen consumption rates of the capped and uncapped gels were measured in the presence of 100 μM NAD^+^ and 10 mM phosphite. Error bars represent the standard deviations of three replicates. (b) Oxygen consumption by the capped gel was monitored in the presence of 100 μM NAD^+^ and 10 mM phosphite (solid line), 100 μM NADH (dashed line), or 100 μM NAD^+^ (dotted line) at 25°C. An arrow indicates the addition of cofactor.

The activity of the gel was evaluated by O_2_ consumption which resulted from the P450cam-catalyzed d-camphor hydroxylation. The capped gel with higher specific surface area displayed a higher initial rate compared to the uncapped gel (Fig. 4a), although the apparent *k_cat_* of the capped gel was significantly reduced compared to PUPPET and an equimolar mixture of PdR, PdX and P450cam (Table 1). A stoichiometric amount of O_2_ consumption was observed in the presence of 100 μM NADH (Fig. 4b). When the reaction was performed with 1 mM d-camphor and 500 μM NADH, the coupling efficiency (d-camphor consumption/NADH consumption) was determined to be 99 ± 1% (Supplementary Fig. S6b and Table S3). Dissolved oxygen was almost completely consumed by the addition of 100 μM NAD^+^ in the presence of 10 mM phosphite, while it was not consumed at all in the absence of phosphite (Fig. 4b).

Interestingly, the initial oxygen consumption rate of both gels in the presence of 100 μM NAD^+^ and phosphite was higher than that in the presence of 100 μM NADH (Fig. 4b and Supplementary Fig. S6a). To understand this phenomenon, the relationship between the initial activity of the capped gel and cofactor concentration was examined. The initial rates apparently followed the Michaelis-Menten equation as a function of the cofactor concentrations (Supplementary Fig. S7). The *K*_m_ for NADH was 3.4 times larger than that for NAD^+^, while the *V*_max_ for NADH was not significantly different from that for NAD^+^ (Table 1). As a result, the catalytic efficiency (*V*_max_/*K*_m_) for NAD^+^ was 2.7 times higher than that for NADH. However, the *K*_m_ for NADH in the d-camphor hydroxylation by free PUPPET was lower than that for NAD^+^ in the reaction with cofactor regeneration by free PTDH, as predicted from an equation which excluded cofactor diffusion (Supplementary Equations).

### Recycling the gel

**Figure 5:**
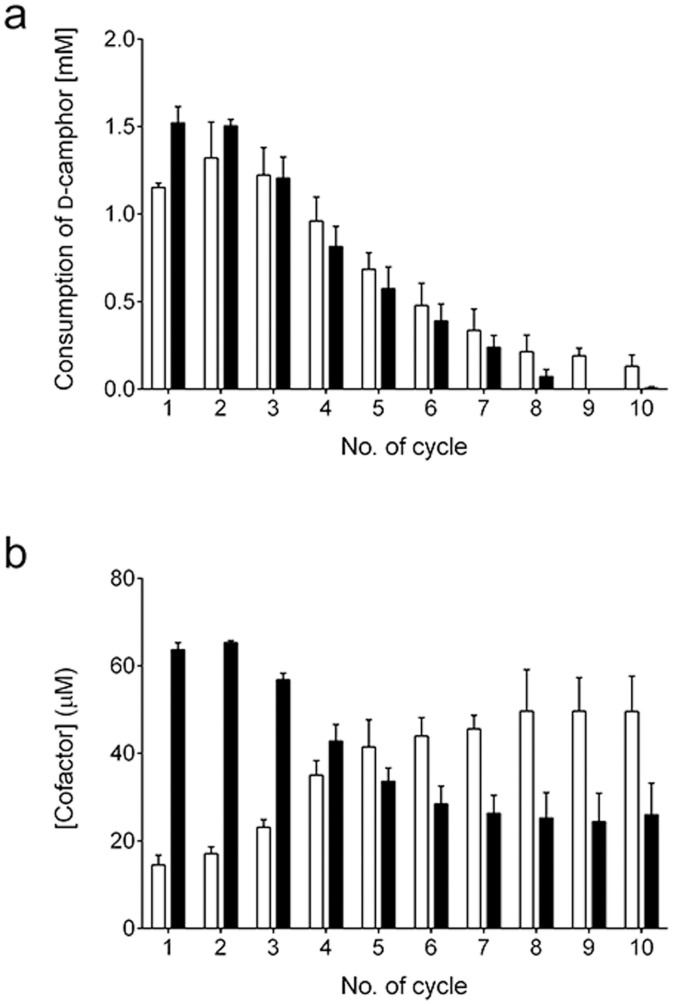
Reusability of the gel. (a) D-Camphor consumption by uncapped (open bar) and capped (closed bar) gels with NADH regeneration. The reaction was conducted in 1.7 mL of 50 mM potassium phosphate buffer, pH 7.4, containing 150 mM potassium chloride, 3 mM d-camphor, 5 mM phosphite, 100 μM NAD^+^ and a gel that was obtained from 30 μL of the mixture containing 80 μM of the three fusion proteins. (b) Concentrations of NADH (open bar) and NAD^+^ (closed bar) in the reaction mixture after each cycle. Error bars represent the standard deviations of three replicates.

To demonstrate the reusability of the gel as an immobilized enzyme, d-camphor hydroxylation was repeated in the presence of 3 mM d-camphor, 100 μM NAD^+^ and 5 mM phosphite (Fig. 5a and Supplementary Fig. S8). Approximately 50% of d-camphor in the reaction mixture was hydroxylated in the first cycle within 20 min, and more than 40% of the d-camphor was still hydroxylated in the second and third cycle. The capped gel hydroxylated larger amounts of d-camphor than the uncapped gel in the first and second cycles, reflecting their slightly higher initial rates. After the third cycle, the consumption of d-camphor gradually decreased for both the uncapped and capped gels. The hydroxylation by the uncapped gel was observed even in the 10th cycle, while the capped gel hydroxylated d-camphor up to 8 cycles. To evaluate the residual activities of the PTDH domain and PUPPET domain in the gel, the cofactors' concentrations in the remaining reaction mixture were determined after each reaction cycle for the uncapped gel (Fig. 5b). The concentration of NADH increased with the increase in number of cycles. This result indicates that the PUPPET domain in the gel lost its activity faster than the PTDH domain.

## Discussion

Herein, we demonstrated the exploitation of a water-insoluble protein gel with a P450 monooxygenase activity, which can be simply obtained by mixing three homodimeric fusion proteins. P450s are known to oxidize a wide range of organic compounds using oxygen with electrons transported from the reducing equivalents to the heme center of P450 by means of redox protein partners^32^. The interest in P450 monooxygenase systems arises not only from their explicit biological significance but also from their industrial potential to catalyze regio/stereo-selective monooxygenation of inert C–H bonds^33^. Typical bacterial P450s, the class I P450s, utilize the auxiliary proteins, ferredoxin reductases (FdRs) and ferredoxins (FdXs); FdRs reduce FdXs with NAD(P)H and reduced FdXs donate electrons to P450s^34^. Direct co-immobilization of FdR, FdX and P450 to form a functional monooxygenation system is almost impossible because FdX's shuttling activity would be impeded by immobilization. Furthermore, the stoichiometric demand of NAD(P)H for a continuous supply of electrons makes *in vitro* large scale applications of P450s unpractical. To overcome these obstacles, we immobilized a *S*. *solfataricus* PCNA-mediated FdR-FdX-P450 complex by the specific cross-linking of PCNA with a homodimeric protein, PTDH, which regenerates NADH with the consumption of phosphite^29^,^30^.

The three PCNA subunits were genetically linked to PTDH and each of the three component proteins of the P450 monooxygenase system. Previously, engineered proteins with multiple interacting domains were reported to self-assemble into a supramolecular network^35^,^36^. The three homodimeric PCNA subunit fusion proteins are able to self-assemble into a supramolecule because the three subunits of *S*. *solfataricus* PCNA exclusively form a heterotrimer without the formation of PCNA2-PCNA3 and PCNA1-PCNA3 heterodimers^24^. However, a mixture of PTDH-PCNA1-PdR, PTDH-PCNA2-PdX and PTDH-PCNA3-P450cam was completely soluble upon addition of buffer (Fig. 2b) because of the high dissociation rate of PCNA3 from the PCNA1-PCNA2 heterodimer^24^.

To prevent the dissociation of the PCNA3 domain, disulfide bonds were introduced at the interfaces between PCNA1 and PCNA3 and between PCNA2 and PCNA3. The wild type *S. solfataricus* PCNA does not possess cysteine residues and we previously demonstrated selective disulfide bond formation by Cys mutagenesis^31^. The equimolar mixture of these cysteine-substituted fusion proteins formed a water-insoluble gel even at lower concentrations (5 μM). Though the yield of gel formation was dependent on the fusion protein concentration added (Fig. 2c), the requirement of micromolar concentrations of fusion proteins is an advantage of our system compared to other protein hydrogels where millimolar concentrations are essential for gel formation^35^,^36^,^37^. As predicted, the gel displayed monooxygenase activity in the presence of NADH as well as in the presence of NAD^+^ and phosphite. While stoichiometric O_2_ consumption was observed in the presence of NADH with high coupling efficiency, approximate complete oxygen consumption by the capped and uncapped gels was observed in the presence of 100 μM NAD^+^ and 10 mM phosphite (Fig. 4b and Supplementary Figure S6). This observation indicates that PTDH regenerated NADH that was consumed by PUPPET-catalyzed d-camphor hydroxylation.

The gel was broken down into smaller particles when PCNA 2_L171C_ and PCNA3_R112C/T180C_ monomers (capping molecules) were added (Fig. 3a). The uncapped gel retained its massive form in the control experiment, indicating that this fragmentation was not due to mechanical breakdown. We postulated that the big mass of gel is an aggregate of smaller gel particles with unbound monomeric PCNA subunits and heterodimeric PCNA1-PCNA2 complex exposed on the surface of these small particles. These exposed domains on one small particle could potentially interact with other exposed domains on neighboring small particles to form multiple bridges between small gel particles when brought into close proximity by centrifugation. Therefore, the uncapped gel remained a huge mass of stable gel even after several cycles of recovery and re-suspension. In the capped gel, the PCNA capping molecules in the vicinity of the exposed monomeric PCNAs could bind complementarily, inhibiting the complex formation between neighboring small particles after centrifugation. This would then lead to dissociation of the small particles upon addition of fresh buffer after removal of supernatant. The capped gel showed an enhanced initial rate in the monooxygenation reaction likely because of the increased specific surface area of the small particles.

The initial O_2_ consumption rate of the capped gel in the presence of 100 μM NAD^+^ and 10 mM phosphite (72.7 μM min^−1^) was 2.8 times higher than that in the presence of 100 μM NADH (25.6 μM min^−1^) (Fig. 4b). The apparent *K*_m_ for NAD^+^ in the presence of phosphite was lower than that for NADH, although the *V*_max_ for NAD^+^ was similar to that for NADH. Thus, this low *K*_m_ value was the reason behind the higher initial rate observed in the presence of NAD^+^ and phosphite. On the other hand, the *K*_m_ value of the free enzyme mixture containing PUPPET and PTDH in a ratio of 1:3 for NAD^+^ in the presence of phosphite was higher than that of free PUPPET for NADH (Table 1). This experimental value is in good agreement with the calculated *K*_m_ value from the kinetic model developed for a free enzyme reaction in which the effect of cofactor diffusion is not considered (Supplementary Equations). This kinetic model suggests that the *K*_m_ for NAD^+^ in the regeneration reaction by the free enzyme mixture is always larger than that of PUPPET for NADH even though the *K*_m_ for NAD^+^ is dependent on the molar ratio of PTDH to PUPPET. Therefore, the lower *K*_m_ value of the capped gel for NAD^+^ compared to that for NADH suggests that diffusion, that is, mass transfer of cofactor, affected the entire reaction catalyzed by the capped gel. In fact, the apparent *K*_m_ value of the capped gel for NADH was increased by 18-fold compared to free PUPPET and this could be the result of low effective concentration of NADH in the gel due to the slow mass-transfer rate of NADH into the gel. The lower apparent *k_cat_* values of capped gel for NADH and NAD^+^ compared to PUPPET and an equimolar mixture of free enzymes also suggest that access of substrates to PUPPET which is buried in the gel may be limited. This is in agreement with previous reports that the kinetic parameters of immobilized enzymes are usually altered compared to those of free enzymes as a result of mass transfer limitation^38^. If the mass-transfer resistance in the gel was high, we speculate that NAD^+^ that was obtained by the PUPPET reaction would be converted to NADH by PTDH in the gel before diffusing out of the gel, and thus, the local concentration of NADH should be higher in the presence of phosphite within the gel. Also, there is a possibility that the properties of PdR were altered by gelation process.

The gel demonstrated reusability as an immobilized enzyme (Fig. 5a). The capped gel hydroxylated larger amounts of d-camphor compared to the uncapped gel in the first and second cycle. This is consistent with the higher initial rate observed for the capped gel. From the third cycle thereafter, the capped gel catalyzed a lower amount of d-camphor hydroxylation compared to the uncapped gel. This is probably a result of more enzyme exposure on the surface of the capped gel leading to faster decline of activity compared to the uncapped gel. Nevertheless, both gels showed a gradual decline in the hydroxylation with an increase in the number of cycles presumably because of enzyme inactivation^20^. To determine the dominating domain which controls the gel's activity, each cofactor concentration was evaluated in the remaining reaction mixture after each cycle of the uncapped gel reaction (Fig. 5b). The increase of NADH concentration with an increasing number of cycles indicates that PUPPET lost its activity faster than PTDH. However, more stable PUPPET complex formed from components of thermostable P450 monooxygenase systems such as a *Thermus thermophilus* monooxygenase system^39^ could overcome the issue of instability. We cannot yet rule out the possibility that the decrease in activity was also a result of loss of some enzymes in the form of gel fibrils during the course of the supernatant removal especially in the smaller particles of the capped gel.

In conclusion, we have developed a simple and viable method for immobilization of a typical bacterial class I P450 enzyme. The P450 monooxygenase activity was reconstituted in a water-insoluble gel by utilizing the self-assembly nature of PTDH-homodimerized *S*. *solfataricus* PCNA subunits. This approach is relatively simple when compared to other related immobilization method such as cross-linked enzyme aggregates (CLEAs). In CLEA approach, enzymes have to be precipitated and then cross-linked by bifunctional reagents such as glutaraldehyde followed by subsequent removal of unreacted cross-linkers. However, in our system, the immobilized enzyme can be easily obtained just by mixing of three fusion proteins in buffer containing oxidized glutathione. Indeed, the gel worked as an immobilized enzyme, exhibiting reusability in its monooxygenation reaction. This water-insoluble gel effectively utilized a cofactor because PTDH, which is an essential component to achieve the water-insoluble gel, regenerated NADH. In the gel without NADH regeneration, slow mass-transfer of the cofactors in the gel reduced the concentration of NADH below what was required to achieve sufficient monooxygenation. This slow mass-transfer in the gel was advantageous in the regeneration system probably because NADH regeneration was superior to cofactor diffusion leading to an increased local NADH concentration. Therefore, our approach should be equally applicable for the immobilization of cofactor-dependent enzymes which require cofactor regeneration for industrial usage and multiple enzymes which are involved in cascade reactions.

## Methods

### Gelation of protein complex

After buffer exchange using a PD SpinTrap G-25 pre-equilibrated with 50 mM potassium phosphate buffer, pH7.4, containing 150 mM potassium chloride, 1 mM d-camphor, and 10 mM oxidized glutathione, equimolar amounts (3 nmol) of the purified PTDH-PCNA1_G108C_-PdR, PTDH-PCNA2_L171C_-PdX and PTDH-PCNA3_R112C/T180C_-P450cam (Supplementary Method) were mixed at various concentrations and incubated overnight at 4°C. After the supernatant was removed by centrifugation at 16, 000×g for 3 min at 4°C and collected, the gels were washed three times with 500 μL of 50 mM potassium phosphate buffer, pH7.4, containing 1 mM d-camphor and all three washes were pooled with the supernatant. The combined wash was then subjected to SDS-PAGE and analyzed using ImageJ to determine the yield. To prepare the capped gel, 200 μL of equal concentrations (80 μM) of PCNA2_L171C_ and PCNA3_R112C/T180C_ in 50 mM potassium phosphate buffer, pH7.4, containing 150 mM potassium chloride, 1 mM d-camphor and 10 mM oxidized glutathione were added to the mixture after the overnight incubation. The samples were rotated at 15 rpm, 4°C for 4 h.

### Activity assays

The phosphite oxidation activity of the PTDH domain in the fusion proteins was determined from NADH production which was monitored by measuring the absorbance of NADH at 340 nm (ε_340_ = 6.22 mM^−1^ cm^−1^). The reaction mixture contained 100 μM NAD^+^, 10 mM phosphite and 90 nM enzyme in 50 mM potassium phosphate buffer, pH7.4, containing 150 mM potassium chloride (buffer A). The activity of the PdR domain in the fusion protein was determined by the reduction of ferricyanide, whose absorbance was monitored at 420 nm (ε_420_ = 1.02 mM^−1^ cm^−1^). The reaction mixture contained 500 μM NADH, 500 μM potassium ferricyanide and 0.5 nM protein in buffer A. A cytochrome c reduction assay was conducted to evaluate the activity of PdX in the fusion protein. The reaction was initiated by adding the C73S/C85S mutant of PdX or PTDH-PCNA2_L171C_-PdX (final concentration = 0.5 nM) into the mixture containing 4 μM PdR, 500 μM NADH and 100 μM cytochrome c in buffer A. The cytochrome c reduction rate was determined based on the absorbance difference between the oxidized and reduced cytochrome c at 550 nm (ε_550_^red^ − ε_550_^ox^ = 21.1 mM^−1^ cm^−1^). The P450cam domain activity was spectroscopically evaluated by the consumption of NADH under the saturated condition. The enzymes, 10 nM P450cam or PTDH-PCNA3_R112C/T180C_-P450cam were added into 4 μM PdR, 50 μM PdX, 1 mM d-camphor and 100 μM NADH in buffer A.

To determine the d-camphor hydroxylation activity of the gels, the oxygen consumption was measured by a Clark-type oxygen electrode. To evaluate the hydroxylation activity with cofactor regeneration, the reaction mixture contained 1 mM d-camphor, 100 μM NAD^+^, 10 mM phosphite and a gel prepared from 30 μL of the mixture containing 80 μM of all three fusion proteins in 1 mL buffer A. To evaluate the activity without cofactor regeneration, the reaction was conducted in the mixture containing 1 mM d-camphor, 100 μM NADH and the gels. For kinetic constant evaluations, the mixture contained 1 mM d-camphor, 30 μL of the mixture containing 80 μM of all three fusion proteins, 0–600 μM NADH or 0–100 μM NAD^+^ and 10 mM phosphite in 1 mL buffer A. All experiments were carried out at 25°C. The least squares fitting of the data to the standard Michaelis-Menten equation was done with GraphPad Prism 6 (GraphPad Software, La Jolla, CA, USA) to estimate *V*_max_ and *K*_m_.

### Determination of coupling efficiency

The mixture contained 1 mM d-camphor, 500 μM NADH and the gel prepared from 30 μL of the mixture containing 80 μM of all three fusion proteins in 1 mL buffer A and was rotated at 30 rpm, 25°C for 20 min and centrifuged at 16, 000×g for 3 min. Remaining NADH was evaluated spectrophotometrically at 340 nm. Remaining d-camphor was extracted from 500 μL of supernatant by 500 μL of CH_2_Cl_2_ containing bromocamphor as an internal standard. After the CH_2_Cl_2_ layer was collected, the aqueous layer was washed with 500 μL CH_2_Cl_2_. The CH_2_Cl_2_ layers were combined and dried with anhydrous sodium sulfate. The dried CH_2_Cl_2_ solution was concentrated under a flow of N_2_ gas and then analyzed by gas chromatography using a Hewlett-Packard 6850 equipped with a flame ionized detector and a HP-1 column.

### Evaluation of reusability

Reactions were initiated by adding 1.7 mL of reaction buffer (50 mM potassium phosphate buffer, pH7.4, containing 150 mM potassium chloride, 3 mM d-camphor, 100 μM NAD^+^ and 5 mM phosphite) to gels, which were prepared from 30 μL of the mixture containing 80 μM fusion proteins and then washed 6 times with 500 μL of buffer A containing 3 mM d-camphor and 5 mM phosphite in advance. The reaction mixture was rotated at 30 rpm, 25°C for 20 min and centrifuged at 16, 000×g for 3 min. After the supernatant was completely removed, 1.7 mL of the fresh reaction buffer was added to the gel again for a subsequent reaction cycle.

The consumption of d-camphor was evaluated by measuring the d-camphor concentration in the supernatant after each reaction cycle by gas chromatography as described above. Meanwhile, the remaining 500 μL of the aqueous layer was used to determine the total cofactor concentration after each cycle for the uncapped gel. Briefly, absorption of the aqueous layer at 340 nm was taken to determine the NADH concentration followed addition of 2 μM of PTDH and 10 mM phosphite (total volume of 1 mL) to the aqueous layer to convert the remaining NAD^+^ to NADH. Absorption at 340 nm was measured after 2 min to allow complete conversion of the cofactor.

## Author Contributions

H.H. conceived the concepts; H.H. and C.Y.T. designed the experiments; C.Y.T. performed the experiments and prepared the first draft. H.H., C.Y.T. and T.N. discussed the results and commented on the manuscript.

## Supplementary Material

Supplementary Information Supplementary Information

## Figures and Tables

**Figure 1:**
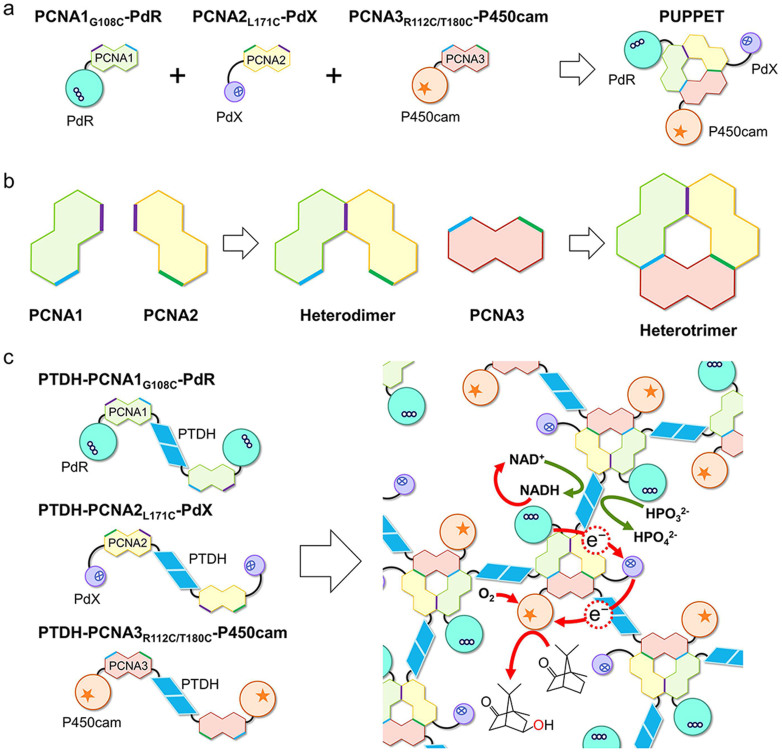
Immobilization of a bacterial cytochrome P450 system by employing self-assembly nature of two oligomeric proteins. (a) PUPPET formed from PCNA1-PdR, PCNA2-PdX and PCNA3-P450cam through *Sulfolobus solfataricus* PCNA self-assembly (b) Step-wise heterotrimerization of *S. solfataricus* PCNA. (c) Supramolecular formation of *S*. *solfataricus* PCNA subunit proteins fused to homodimeric PTDH. PTDH oxidizes phosphite to phosphate with concomitant reduction of NAD^+^. The resultant NADH supplies electrons to PdR in the PUPPET domain to be transferred to P450cam via PdX.

**Table 1:** Kinetic constants of the capped gel, PTDH and PUPPET for the cofactors

Enzyme	Cofactor	*V*_max_ (μM min^−1^)	*K*_m_ (μM)	*V*_max_/*K*_m_ (min^−1^)	Apparent *k_cat_* (min^−1^)
Capped gel^a^	NADH	105 ± 12	164 ± 51	0.640 ± 0.21	47 ± 5
	NAD^+^	82 ± 18	48 ± 25	1.71 ± 0.10	36 ± 8
PUPPET^b^	NADH	46.5 ± 1.0	9.0 ± 0.7	5.17 ± 0.42	517 ± 11
PTDH^c^	NAD^+^	22.9 ± 0.5	59.0 ± 3.2	0.39 ± 0.02	254 ± 5
PUPPET:PTDH = 1:3^d^	NAD^+^	51.1 ± 1.0 (46.5)	38.2 ± 1.6 (39.3)	1.34 ± 0.06 (1.18)	568 ± 11^e^
PdR:PdX:P450cam = 1:1:1^f^	NADH	(1.640 ± 0.12) × 10^3^	240 ± 47	6.9 ± 1.4	164 ± 12

^a^The apparent *k_cat_* for capped gel was estimated based on the gel yield as 94%.

^b^The reaction mixture contained various concentrations of NADH, 1 mM D-camphor and 90 nM PUPPET in 50 mM potassium phosphate buffer, pH7.4, containing 150 mM potassium chloride (buffer A).

^c^The reaction mixture contained various concentrations of NAD^+^, 10 mM phosphite and 90 nM PTDH in buffer A.

^d^Values in brackets show kinetic parameters obtained by substituting *k_cat_* and *K_m_* values of free PUPPET and PTDH into the Supporting Equations when the reaction mixture contains 90 nM PUPPET and 270 nM PTDH.

^e^The apparent *k_cat_* was estimated from PUPPET's concentration.

^f^The reaction mixture contained various concentrations of NADH, 1 mM D-camphor and an equimolar concentration (10 μM) of PdR, PdX and P450cam in buffer A.
