# Supplementary material for: Supramolecular protein assembly supports immobilization of a cytochrome P450 monooxygenase system as water-insoluble gel
Source: Sci Rep. 2015 Mar 3;5:8648. doi: 10.1038/srep08648 (PMC4346803; doi:10.1038/srep08648)
Supplement: Supplementary Information [file srep08648-s1.doc]

**Supplementary Information**

Supramolecular protein assembly supports immobilization of a cytochrome P450 monooxygenase system as water-insoluble gel

Cheau Yuaan Tan^1^, Hidehiko Hirakawa^2*^, Teruyuki Nagamune^1,2,*^

^1^Department of Bioengineering, School of Engineering, The University of Tokyo, 7-3-1 Hongo, Bunkyo-ku, Tokyo 113-8656, Japan

^2^Department of Chemistry and Biotechnology, School of Engineering, The University of Tokyo, 7-3-1 Hongo, Bunkyo-ku, Tokyo 113-8656, Japan

*Corresponding authors: Telephone: +81-3-5841-7356, Fax: +81-3-5841-8657, E-mail: hirakawa@bio.t.u-tokyo.ac.jp, nagamune@bioeng.t.u-tokyo.ac.jp

**Supplementary Methods**

***Materials*** NADH and NAD^+^ were purchased from Oriental Yeast Co. Ltd. (Tokyo, Japan). Disodium hydrogen phosphite pentahydrate, d-camphor, oxidized glutathione and aminolevulinic acid hydrochloride were purchased from Wako Pure Chemical Industries (Osaka, Japan). *Escherichia coli* BL21 Star (DE3) was purchased from Invitrogen (Carlsbad, CA, USA). *E. coli* T7 Express *I*^q^ was purchased from New England Biolabs (Ipswich, MA, USA). HisTrap FF crude column (1.6×2.5 cm), HiTrap Q HP column (1.6×2.5 cm), HiLoad 16/60 Superdex 75 pg column (1.6×60 cm), HiLoad 16/60 Superdex 200 pg column (1.6×60 cm) and PD SpinTrap G-25 were procured from GE Healthcare (Little Chalfont, UK).

***Construction of expression vectors*** The gene encoding the PCNA1-PdR fusion protein was amplified from pET28b+P1R ^[S1]^ by PCR using two primers, 5’-GGAATTCGCTCTTCAATG TTTAAAATTGTGTATCCGAACGC-3’ and 5’-CGGGATCCTCAGGCACTACTCAGTTCA G-3’. The generated DNA fragment was digested with *Sap*I and *Bam*HI, and then ligated into pPTDHsap ^[S2]^ that was digested with the same restriction enzymes. The resulting plasmid, pPD1R, expressed the PTDH-PCNA1-PdR fusion proteins. The plasmid, pPD3C, which expressed the PTDH-PCNA3-P450cam fusion protein, was constructed as above, except the gene encoding the PCNA3-P450cam fusion protein was amplified from pET15b+P3MC ^[S2]^ by PCR using two primers, 5’-GGAATTCGCTCTTCAATGATCTACCTGAAATCTTTCGA AC-3’ and 5’-CGGGATCCTTATACCGCTTTGGTAGTCGC-3’. The G108C mutation in PCNA1 of PTDH-PCNA1-PdR, the L171C mutation in PCNA2 of PTDH-PCNA2-PdX and the R112C/T180C mutations in PCNA3 of PTDH-PCNA3-P450cam were introduced as reported previously ^[S1]^, except that pPD1R, pPD2X and pPD3C were used as templates for the PCR-based mutagenesis, respectively. The resulting plasmids, pPD1cR, pPD2cX and pPD3cC, expressed PTDH-PCNA1_G108C_-PdR, PTDH-PCNA2_L171C_-PdX and PTDH-PCNA3_R112C/T180C_-P450cam, respectively.

***Protein expression and purification.*** BL21 star (DE3) cells transformed with pPD1cR were inoculated into 5 ml LB medium containing 0.1 mg/ml Amp and 1% glucose. The pre-culture was transferred into 1 L TB medium and the cells were grown at 37°C until the OD_600_ reached approximately 0.6. Then, protein expression was induced by 1 mM IPTG and the culture was continued at 20°C overnight. The cells expressing PTDH-PCNA1_G108C_-PdR were harvested by centrifugation at 6000×g for 20 min and stored at -20°C until use. PTDH-PCNA1-PdR was expressed as described above. PTDH-PCNA2-PdX and PTDH-PCNA2_L171C_-PdX were expressed as above for PTDH-PCNA1_G108C_-PdR, except that T7 Express *I*^q^ was transformed with pPD2X and pPD2cX, respectively. PTDH-PCNA3-P450cam and PTDH-PCNA3_R112C/T180C_-P450cam were expressed as above for PTDH-PCNA1_G108C_-PdR, except that T7 Express *I*^q^ was transformed with pPD3C and pPD3cC, respectively and the proteins were expressed at 27°C in TB media containing 1 mM 5-aminolevulic acid.

Cells expressing PTDH-PCNA1_G108C_-PdR were resuspended with 20 mM potassium phosphate buffer, pH 7.4, containing 150 mM potassium chloride, 1 mM DTT and 10 mM imidazole and disrupted by ultrasonication. After the cell debris was removed by centrifugation at 22,000×g for 30 min, the supernatant was applied to a HisTrap FF crude column. After washing the column with the buffer, the protein was eluted with a linear gradient of imidazole (10 – 200 mM) over 10 column volumes (CVs). The eluted protein was diluted 3-fold in 20 mM potassium phosphate buffer, pH 7.4, containing 5 mM DTT and then applied to a HiTrapQ HP column pre-equilibrated with 20 mM potassium phosphate buffer, pH 7.4, containing 5 mM DTT and 50 mM potassium chloride. After washing the column with the buffer, the protein was eluted with a linear gradient of potassium chloride (50-500 mM) over 10 CVs. Fractions containing the protein were pooled and concentrated with an Amicon Ultra-15 Centrifugal Unit (50,000 NMWL). The concentrated protein was subjected to size-exclusion chromatography on a HiLoad 16/60 Superdex 200 column pre-equilibrated with 50 mM potassium phosphate buffer, pH 7.4, containing 10 mM DTT and 150 mM potassium chloride. Fractions with a ratio of Abs_280_/Abs_455_ less than 10 were collected and concentrated with the Amicon centrifugal unit. The concentration of PTDH-PCNA1_G108C_-PdR was calculated by using the molar extinction coefficient of PCNA1-PdR (ε_455_ = 11.0 mM^−1^cm^−1^) ^[S3]^. PTDH-PCNA1-PdR and PTDH-PCNA2-PdX was purified as described above. PTDH-PCNA2_L171C_-PdX was purified as described above for PTDH-PCNA1_G108C_-PdR, except that the cell lysates were applied to a DE52 column (2.5×11 cm) pre-equilibrated with 20 mM potassium phosphate buffer, pH 7.4, containing 1 mM DTT and 150 mM potassium chloride and the proteins were eluted with a linear gradient of potassium chloride (150-500 mM) over 10 CVs prior to affinity chromatography using HisTrap FF crude column. Fractions with a ratio of Abs_412_/Abs_280_ higher than 0.15 were pooled after anion exchange chromatography and size-exclusion chromatography was not performed. The concentrations of PTDH-PCNA2_L171C_-PdX and PTDH-PCNA2-PdX were calculated by using the molar extinction coefficient of PdX (ε_412_ = 11.0 mM^−1^cm^−1^) ^[S4]^. PTDH-PCNA3_R112C/T180C_-P450cam and PTDH-PCNA3-P450cam were purified as described above for PTDH-PCNA1_G108C_-PdR, except that the buffers contained 1 mM d-camphor and the fractions with a ratio of Abs_392_/Abs_280_ higher than 0.9 were collected after anion exchange chromatography and size-exclusion chromatography. The protein concentrations were determined from the extinction coefficient ε_392_ = 90.2 mM^−1^cm^−1 [S3]^. PCNA2_L171C_ and PCNA3_R112C/T180C_ were expressed and purified as reported previously ^[S1]^.

**Protein Sequences**

PdR

MNANDNVVIVGTGLAGVEVAFGLRASGWEGNIRLVGDATVIPHHLPPLSKAYLAGKATAESLYLRTPDAYAAQNIQLLGGTQVTAINRDRQQVILSDGRALDYDRLVLATGGRPRPLPVASGAVGKANNFRYLRTLEDAECIRRQLIADNRLVVIGGGYIGLEVAATAIKANMHVTLLDTAARVLERVTAPPVSAFYEHLHREAGVDIRTGTQVCGFEMSTDQQKVTAVLCEDGTRLPADLVIAGIGLIPNCELASAAGLQVDNGIVINEHMQTSDPLIMAVGDCARFHSQLYDRWVRIESVPNALEQARKIAAILCGKVPRDEAAPWFWSDQYEIGLKMVGLSEGYDRIIVRGSLAQPDFSVFYLQGDRVLAVDTVNRPVEFNQSKQIITDRLPVEPNLLGDESVPLKEIIAAAKAELSSA

The C73S/C85S mutant of PdX

MSKVVYVSHDGTRRELDVADGVSLMQAAVSNGIYDIVGDCGGSASCATCHVYVNEAFTDKVPAANEREIGMLESVTAELKPNSRLSCQIIMTPELDGIVVDVPDRQW

P450cam

MTTETIQSNANLAPLPPHVPEHLVFDFDMYNPSNLSAGVQEAWAVLQESNVPDLVWTRCNGGHWIATRGQLIREAYEDYRHFSSECPFIPREAGEAYDFIPTSMDPPEQRQFRALANQVVGMPVVDKLENRIQELACSLIESLRPQGQCNFTEDYAEPFPIRIFMLLAGLPEEDIPHLKYLTDQMTRPDGSMTFAEAKEALYDYLIPIIEQRRQKPGTDAISIVANGQVNGRPITSDEAKRMCGLLLVGGLDTVVNFLSFSMEFLAKSPEHRQELIERPERIPAACEELLRRFSLVADGRILTSDYEFHGVQLKKGDQILLPQMLSGLDERENACPMHVDFSRQKVSHTTFGHGSHLCLGQHLARREIIVTLKEWLTRIPDFSIAPGAQIQHKSGIVSGVQALPLVWDPATTKAV

The E175A/A176R mutant of PTDH

MLPKLVITHRVHDEILQLLAPHCELMTNQTDSTLTREEILRRCRDAQAMMAFMPDRVDADFLQACPELRVVGCALKGFDNFDVDACTARGVWLTFVPDLLTVPTAELAIGLAVGLGRHLRAADAFVRSGEFQGWQPQFYGTGLDNATVGILGMGAIGLAMADRLQGWGATLQYHARKALDTQTEQRLGLRQVACSELFASSDFILLALPLNADTQHLVNAELLALVRPGALLVNPCRGSVVDEAAVLAALERGQLGGYAADVFEMEDWARADRPRLIDPALLAHPNTLFTPHIGSAVRAVRLEIERCAAQNIIQVLAGARPINAANRLPKAEPAAC

PCNA2_L171C_

MMKAKVIDAVSFSYILRTVGDFLSEANFIVTKEGIRVSGIDPSRVVFLDIFLPSSYFEGFEVSQEKEIIGFKLEDVNDILKRVLKDDTLILSSNESKLTLTFDGEFTRSFELPLIQVESTQPPSVNLEFPFKAQLLTITFADIIDELSDLGEVLNIHSKENKLYFEVIGDCSTAKVELSTDNGTLLEASGADVSSSYGMEYVANTTKMRRASDSMELYFGSQIPLKLRFKLPQEGYGDFYIAPRAD

PCNA3_R112C/T180C_

MIYLKSFERNIRLINMKVVYDDVRVLKDIIQALARLVDEAVLKFKQDSVELVALDRAHISLISVNLPREMFKEYDVNDEFKFGFNTQYLMKILKVAKRKEAIEIASESPDSVIINIIGSTNREFNVCNLEVSEQEIPEINLQFDISATISSDGFKSAISEVSTVTDNVVVEGHEDRILIKAEGESEVEVEFSKDCGGLQDLEFSKESKNSYSAEYLDDVLSLTKLSDYVKISFGNQKPLQLFFNMEGGGKVTYLLAPKV

PCNA1_G108C_-PdR

MFKIVYPNAKDFFSFINSITNVTDSIILNFTEDGIFSRHLTEDKVLMAIMRIPKDVLSEYSIDSPTSVKLDVSSVKKILSKASSKKATIELTETDSGLKIIIRDEKSCAKSTIYIKAEKGQVEQLTEPKVNLAVNFTTDESVLNVIAADVTLVGEEMRISTEEDKIKIEAGEEGKRYVAFLMKDKPLKELSIDTSASSSYSAEMFKDAVKGLRGFSAPTMVSFGENLPMKIDVEAVSGGHMIFWIAPRLGGGGSGGGGSMNANDNVVIVGTGLAGVEVAFGLRASGWEGNIRLVGDATVIPHHLPPLSKAYLAGKATAESLYLRTPDAYAAQNIQLLGGTQVTAINRDRQQVILSDGRALDYDRLVLATGGRPRPLPVASGAVGKANNFRYLRTLEDAECIRRQLIADNRLVVIGGGYIGLEVAATAIKANMHVTLLDTAARVLERVTAPPVSAFYEHLHREAGVDIRTGTQVCGFEMSTDQQKVTAVLCEDGTRLPADLVIAGIGLIPNCELASAAGLQVDNGIVINEHMQTSDPLIMAVGDCARFHSQLYDRWVRIESVPNALEQARKIAAILCGKVPRDEAAPWFWSDQYEIGLKMVGLSEGYDRIIVRGSLAQPDFSVFYLQGDRVLAVDTVNRPVEFNQSKQIITDRLPVEPNLLGDESVPLKEIIAAAKAELSSA

PCNA2_L171C_-PdX

MMKAKVIDAVSFSYILRTVGDFLSEANFIVTKEGIRVSGIDPSRVVFLDIFLPSSYFEGFEVSQEKEIIGFKLEDVNDILKRVLKDDTLILSSNESKLTLTFDGEFTRSFELPLIQVESTQPPSVNLEFPFKAQLLTITFADIIDELSDLGEVLNIHSKENKLYFEVIGDCSTAKVELSTDNGTLLEASGADVSSSYGMEYVANTTKMRRASDSMELYFGSQIPLKLRFKLPQEGYGDFYIAPRADGGGGSLVPRGSGGGGSMSKVVYVSHDGTRRELDVADGVSLMQAAVSNGIYDIVGDCGGSASCATCHVYVNEAFTDKVPAANEREIGMLESVTAELKPNSRLSCQIIMTPELDGIVVDVPDRQW

PCNA3_R112C/T180C_-P450cam

MIYLKSFERNIRLINMKVVYDDVRVLKDIIQALARLVDEAVLKFKQDSVELVALDRAHISLISVNLPREMFKEYDVNDEFKFGFNTQYLMKILKVAKRKEAIEIASESPDSVIINIIGSTNREFNVCNLEVSEQEIPEINLQFDISATISSDGFKSAISEVSTVTDNVVVEGHEDRILIKAEGESEVEVEFSKDCGGLQDLEFSKESKNSYSAEYLDDVLSLTKLSDYVKISFGNQKPLQLFFNMEGGGKVTYLLAPKVGGSMTTETIQSNANLAPLPPHVPEHLVFDFDMYNPSNLSAGVQEAWAVLQESNVPDLVWTRCNGGHWIATRGQLIREAYEDYRHFSSECPFIPREAGEAYDFIPTSMDPPEQRQFRALANQVVGMPVVDKLENRIQELACSLIESLRPQGQCNFTEDYAEPFPIRIFMLLAGLPEEDIPHLKYLTDQMTRPDGSMTFAEAKEALYDYLIPIIEQRRQKPGTDAISIVANGQVNGRPITSDEAKRMCGLLLVGGLDTVVNFLSFSMEFLAKSPEHRQELIERPERIPAACEELLRRFSLVADGRILTSDYEFHGVQLKKGDQILLPQMLSGLDERENACPMHVDFSRQKVSHTTFGHGSHLCLGQHLARREIIVTLKEWLTRIPDFSIAPGAQIQHKSGIVSGVQALPLVWDPATTKAV

PTDH-PCNA1-PdR

MLPKLVITHRVHDEILQLLAPHCELMTNQTDSTLTREEILRRCRDAQAMMAFMPDRVDADFLQACPELRVVGCALKGFDNFDVDACTARGVWLTFVPDLLTVPTAELAIGLAVGLGRHLRAADAFVRSGEFQGWQPQFYGTGLDNATVGILGMGAIGLAMADRLQGWGATLQYHARKALDTQTEQRLGLRQVACSELFASSDFILLALPLNADTQHLVNAELLALVRPGALLVNPCRGSVVDEAAVLAALERGQLGGYAADVFEMEDWARADRPRLIDPALLAHPNTLFTPHIGSAVRAVRLEIERCAAQNIIQVLAGARPINAANRLPKAEPAACSSGLPATGGGSMFKIVYPNAKDFFSFINSITNVTDSIILNFTEDGIFSRHLTEDKVLMAIMRIPKDVLSEYSIDSPTSVKLDVSSVKKILSKASSKKATIELTETDSGLKIIIRDEKSGAKSTIYIKAEKGQVEQLTEPKVNLAVNFTTDESVLNVIAADVTLVGEEMRISTEEDKIKIEAGEEGKRYVAFLMKDKPLKELSIDTSASSSYSAEMFKDAVKGLRGFSAPTMVSFGENLPMKIDVEAVSGGHMIFWIAPRLGGGGSGGGGSMNANDNVVIVGTGLAGVEVAFGLRASGWEGNIRLVGDATVIPHHLPPLSKAYLAGKATAESLYLRTPDAYAAQNIQLLGGTQVTAINRDRQQVILSDGRALDYDRLVLATGGRPRPLPVASGAVGKANNFRYLRTLEDAECIRRQLIADNRLVVIGGGYIGLEVAATAIKANMHVTLLDTAARVLERVTAPPVSAFYEHLHREAGVDIRTGTQVCGFEMSTDQQKVTAVLCEDGTRLPADLVIAGIGLIPNCELASAAGLQVDNGIVINEHMQTSDPLIMAVGDCARFHSQLYDRWVRIESVPNALEQARKIAAILCGKVPRDEAAPWFWSDQYEIGLKMVGLSEGYDRIIVRGSLAQPDFSVFYLQGDRVLAVDTVNRPVEFNQSKQIITDRLPVEPNLLGDESVPLKEIIAAAKAELSSA

PTDH-PCNA2-PdX

MLPKLVITHRVHDEILQLLAPHCELMTNQTDSTLTREEILRRCRDAQAMMAFMPDRVDADFLQACPELRVVGCALKGFDNFDVDACTARGVWLTFVPDLLTVPTAELAIGLAVGLGRHLRAADAFVRSGEFQGWQPQFYGTGLDNATVGILGMGAIGLAMADRLQGWGATLQYHARKALDTQTEQRLGLRQVACSELFASSDFILLALPLNADTQHLVNAELLALVRPGALLVNPCRGSVVDEAAVLAALERGQLGGYAADVFEMEDWARADRPRLIDPALLAHPNTLFTPHIGSAVRAVRLEIERCAAQNIIQVLAGARPINAANRLPKAEPAACSSGLPATGGGSMMKAKVIDAVSFSYILRTVGDFLSEANFIVTKEGIRVSGIDPSRVVFLDIFLPSSYFEGFEVSQEKEIIGFKLEDVNDILKRVLKDDTLILSSNESKLTLTFDGEFTRSFELPLIQVESTQPPSVNLEFPFKAQLLTITFADIIDELSDLGEVLNIHSKENKLYFEVIGDLSTAKVELSTDNGTLLEASGADVSSSYGMEYVANTTKMRRASDSMELYFGSQIPLKLRFKLPQEGYGDFYIAPRADGGGGSLVPRGSGGGGSMSKVVYVSHDGTRRELDVADGVSLMQAAVSNGIYDIVGDCGGSASCATCHVYVNEAFTDKVPAANEREIGMLESVTAELKPNSRLSCQIIMTPELDGIVVDVPDRQW

PTDH-PCNA3-P450cam

MLPKLVITHRVHDEILQLLAPHCELMTNQTDSTLTREEILRRCRDAQAMMAFMPDRVDADFLQACPELRVVGCALKGFDNFDVDACTARGVWLTFVPDLLTVPTAELAIGLAVGLGRHLRAADAFVRSGEFQGWQPQFYGTGLDNATVGILGMGAIGLAMADRLQGWGATLQYHARKALDTQTEQRLGLRQVACSELFASSDFILLALPLNADTQHLVNAELLALVRPGALLVNPCRGSVVDEAAVLAALERGQLGGYAADVFEMEDWARADRPRLIDPALLAHPNTLFTPHIGSAVRAVRLEIERCAAQNIIQVLAGARPINAANRLPKAEPAACSSGLPATGGGSMIYLKSFERNIRLINMKVVYDDVRVLKDIIQALARLVDEAVLKFKQDSVELVALDRAHISLISVNLPREMFKEYDVNDEFKFGFNTQYLMKILKVAKRKEAIEIASESPDSVIINIIGSTRREFNVRNLEVSEQEIPEINLQFDISATISSDGFKSAISEVSTVTDNVVVEGHEDRILIKAEGESEVEVEFSKDTGGLQDLEFSKESKNSYSAEYLDDVLSLTKLSDYVKISFGNQKPLQLFFNMEGGGKVTYLLAPKVGGSMTTETIQSNANLAPLPPHVPEHLVFDFDMYNPSNLSAGVQEAWAVLQESNVPDLVWTRCNGGHWIATRGQLIREAYEDYRHFSSECPFIPREAGEAYDFIPTSMDPPEQRQFRALANQVVGMPVVDKLENRIQELACSLIESLRPQGQCNFTEDYAEPFPIRIFMLLAGLPEEDIPHLKYLTDQMTRPDGSMTFAEAKEALYDYLIPIIEQRRQKPGTDAISIVANGQVNGRPITSDEAKRMCGLLLVGGLDTVVNFLSFSMEFLAKSPEHRQELIERPERIPAACEELLRRFSLVADGRILTSDYEFHGVQLKKGDQILLPQMLSGLDERENACPMHVDFSRQKVSHTTFGHGSHLCLGQHLARREIIVTLKEWLTRIPDFSIAPGAQIQHKSGIVSGVQALPLVWDPATTKAV

PTDH-PCNA1_G108C_-PdR

MLPKLVITHRVHDEILQLLAPHCELMTNQTDSTLTREEILRRCRDAQAMMAFMPDRVDADFLQACPELRVVGCALKGFDNFDVDACTARGVWLTFVPDLLTVPTAELAIGLAVGLGRHLRAADAFVRSGEFQGWQPQFYGTGLDNATVGILGMGAIGLAMADRLQGWGATLQYHARKALDTQTEQRLGLRQVACSELFASSDFILLALPLNADTQHLVNAELLALVRPGALLVNPCRGSVVDEAAVLAALERGQLGGYAADVFEMEDWARADRPRLIDPALLAHPNTLFTPHIGSAVRAVRLEIERCAAQNIIQVLAGARPINAANRLPKAEPAACSSGLPATGGGSMFKIVYPNAKDFFSFINSITNVTDSIILNFTEDGIFSRHLTEDKVLMAIMRIPKDVLSEYSIDSPTSVKLDVSSVKKILSKASSKKATIELTETDSGLKIIIRDEKSCAKSTIYIKAEKGQVEQLTEPKVNLAVNFTTDESVLNVIAADVTLVGEEMRISTEEDKIKIEAGEEGKRYVAFLMKDKPLKELSIDTSASSSYSAEMFKDAVKGLRGFSAPTMVSFGENLPMKIDVEAVSGGHMIFWIAPRLGGGGSGGGGSMNANDNVVIVGTGLAGVEVAFGLRASGWEGNIRLVGDATVIPHHLPPLSKAYLAGKATAESLYLRTPDAYAAQNIQLLGGTQVTAINRDRQQVILSDGRALDYDRLVLATGGRPRPLPVASGAVGKANNFRYLRTLEDAECIRRQLIADNRLVVIGGGYIGLEVAATAIKANMHVTLLDTAARVLERVTAPPVSAFYEHLHREAGVDIRTGTQVCGFEMSTDQQKVTAVLCEDGTRLPADLVIAGIGLIPNCELASAAGLQVDNGIVINEHMQTSDPLIMAVGDCARFHSQLYDRWVRIESVPNALEQARKIAAILCGKVPRDEAAPWFWSDQYEIGLKMVGLSEGYDRIIVRGSLAQPDFSVFYLQGDRVLAVDTVNRPVEFNQSKQIITDRLPVEPNLLGDESVPLKEIIAAAKAELSSA

PTDH-PCNA2_L171C_-PdX

MLPKLVITHRVHDEILQLLAPHCELMTNQTDSTLTREEILRRCRDAQAMMAFMPDRVDADFLQACPELRVVGCALKGFDNFDVDACTARGVWLTFVPDLLTVPTAELAIGLAVGLGRHLRAADAFVRSGEFQGWQPQFYGTGLDNATVGILGMGAIGLAMADRLQGWGATLQYHARKALDTQTEQRLGLRQVACSELFASSDFILLALPLNADTQHLVNAELLALVRPGALLVNPCRGSVVDEAAVLAALERGQLGGYAADVFEMEDWARADRPRLIDPALLAHPNTLFTPHIGSAVRAVRLEIERCAAQNIIQVLAGARPINAANRLPKAEPAACSSGLPATGGGSMMKAKVIDAVSFSYILRTVGDFLSEANFIVTKEGIRVSGIDPSRVVFLDIFLPSSYFEGFEVSQEKEIIGFKLEDVNDILKRVLKDDTLILSSNESKLTLTFDGEFTRSFELPLIQVESTQPPSVNLEFPFKAQLLTITFADIIDELSDLGEVLNIHSKENKLYFEVIGDCSTAKVELSTDNGTLLEASGADVSSSYGMEYVANTTKMRRASDSMELYFGSQIPLKLRFKLPQEGYGDFYIAPRADGGGGSLVPRGSGGGGSMSKVVYVSHDGTRRELDVADGVSLMQAAVSNGIYDIVGDCGGSASCATCHVYVNEAFTDKVPAANEREIGMLESVTAELKPNSRLSCQIIMTPELDGIVVDVPDRQW

PTDH-PCNA3_R112C/T180C_-P450cam

MLPKLVITHRVHDEILQLLAPHCELMTNQTDSTLTREEILRRCRDAQAMMAFMPDRVDADFLQACPELRVVGCALKGFDNFDVDACTARGVWLTFVPDLLTVPTAELAIGLAVGLGRHLRAADAFVRSGEFQGWQPQFYGTGLDNATVGILGMGAIGLAMADRLQGWGATLQYHARKALDTQTEQRLGLRQVACSELFASSDFILLALPLNADTQHLVNAELLALVRPGALLVNPCRGSVVDEAAVLAALERGQLGGYAADVFEMEDWARADRPRLIDPALLAHPNTLFTPHIGSAVRAVRLEIERCAAQNIIQVLAGARPINAANRLPKAEPAACSSGLPATGGGSMIYLKSFERNIRLINMKVVYDDVRVLKDIIQALARLVDEAVLKFKQDSVELVALDRAHISLISVNLPREMFKEYDVNDEFKFGFNTQYLMKILKVAKRKEAIEIASESPDSVIINIIGSTRREFNVCNLEVSEQEIPEINLQFDISATISSDGFKSAISEVSTVTDNVVVEGHEDRILIKAEGESEVEVEFSKDCGGLQDLEFSKESKNSYSAEYLDDVLSLTKLSDYVKISFGNQKPLQLFFNMEGGGKVTYLLAPKVGGSMTTETIQSNANLAPLPPHVPEHLVFDFDMYNPSNLSAGVQEAWAVLQESNVPDLVWTRCNGGHWIATRGQLIREAYEDYRHFSSECPFIPREAGEAYDFIPTSMDPPEQRQFRALANQVVGMPVVDKLENRIQELACSLIESLRPQGQCNFTEDYAEPFPIRIFMLLAGLPEEDIPHLKYLTDQMTRPDGSMTFAEAKEALYDYLIPIIEQRRQKPGTDAISIVANGQVNGRPITSDEAKRMCGLLLVGGLDTVVNFLSFSMEFLAKSPEHRQELIERPERIPAACEELLRRFSLVADGRILTSDYEFHGVQLKKGDQILLPQMLSGLDERENACPMHVDFSRQKVSHTTFGHGSHLCLGQHLARREIIVTLKEWLTRIPDFSIAPGAQIQHKSGIVSGVQALPLVWDPATTKAV

*Peptide linkers are highlighted by yellow. C73S/C85S mutations in PdX, E175A/A176R mutations in PTDH and cysteine mutations into the PCNA subunits are indicated by red letters.

**Tables**

**Supplementary Table S1. Molecular masses of homodimeric fusion proteins determined by size exclusion chromatography (SEC) and their respective ratio compared with PTDH-PCNA2-PdX**

| Protein | Molecular mass of homodimers (kDa) | |  | Ratio | |
| --- | --- | --- | --- | --- | --- |
|  | Calculated | SEC |  | Calculated | SEC |
| PTDH-PCNA1_G108C_-PdR | 220 | 480 |  | 1.43 | 1.3 |
| PTDH-PCNA2_L171C_-PdX | 154 | 400 |  | 1.00 | 1.1 |
| PTDH-PCNA3_R112C/T180C_-P450cam | 226 | 540 |  | 1.47 | 1.5 |
| PTDH-PCNA2-PdX | 154 | 370 |  | 1.00 | 1.0 |

**Supplementary Table S2.** Initial activities of PTDH, PdR, PdX and P450cam domains in fusion proteins.

| Protein | | Initial rate (µM min^-1^) |
| --- | --- | --- |
| PTDH-PCNA1_G108C_-PdR | PTDH domain [a] | 14.6 ± 0.03 |
|  | PdR domain [b] | 41.3 ± 0.15 |
| PTDH-PCNA2_L171C_-PdX | PTDH domain [a] | 17.2 ± 0.09 |
|  | PdX domain [c] | 6.7 ± 0.06 |
| PTDH-PCNA3_R112C/T180C_-P450cam | PTDH domain [a] | 15.1 ± 0.11 |
|  | P450cam domain [d] | 20.1 ± 0.30 |
| PTDH [a] | | 15.0 ± 0.18 |
| PdR [b] | | 41.8 ± 0.73 |
| PdX [c] | | 6.5 ± 0.09 |
| P450cam [d] | | 19.6 ± 0.21 |

[a] NAD^+^ reduction activities of PTDH were detemined by monitoring the absorption at 340 nm. The reaction mixture contained 100 µM NAD^+^, 10 mM phosphite and 90 nM proteins in 50 mM potassium phosphate buffer, pH 7.4, containing 150 mM potassium chloride. [b] Ferricyanide reduction activities of PdR were determined by monitoring absorption at 420 nm. The reaction mixture contained 500 µM NADH, 500 µM potassium ferricyanide and 0.5 nM protein in the buffer above. [c] Electron donation activities of PdX to cytochrome c were determined by monitoring absorption at 550 nm. The reaction mixture contained 0.5 nM PdX or PTDH-PCNA2_L171C_-PdX, 4 µM PdR, 500 µM NADH and 100 µM cytochrome c in the buffer above. [d] P450cam domain activity was determined by monitoring NADH consumption under the saturated condition. The reaction mixture contained 10 nM P450cam or PTDH-PCNA3_R112C/T180C_-P450cam, 4 µM PdR, 50 µM PdX, 100 µM NADH and 1 mM d-camphor in the buffer above. All experiments were performed at 25°C.

**Supplementary Table S3.** **Coupling efficiency of capped gel after 20 min reaction.**

|  | D-camphor consumption (mM) |  | NADH consumption (mM) | Coupling efficiency (%) |
| --- | --- | --- | --- | --- |
|  |
| Capped gel | 0.369 ± 0.04 |  | 0.372 ± 0.04 | 99 ± 1 |

**Figures**

**a**


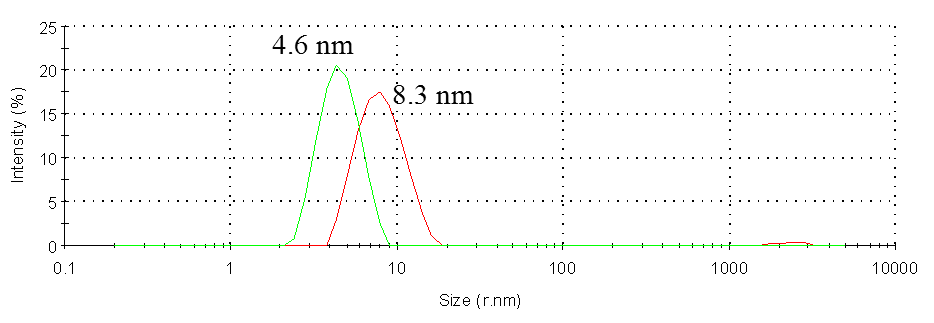


**b**


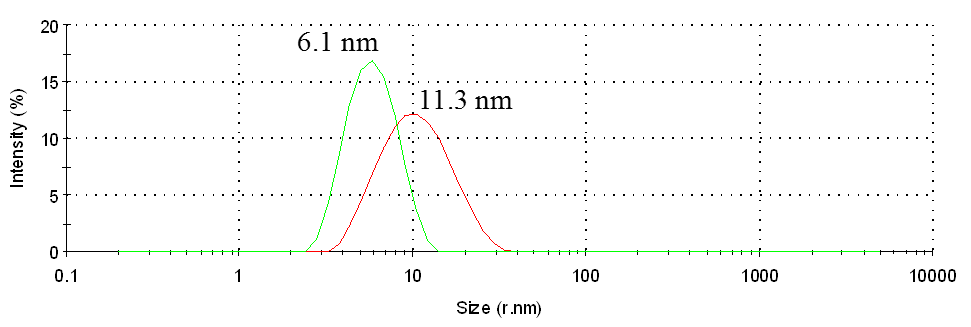


**c**


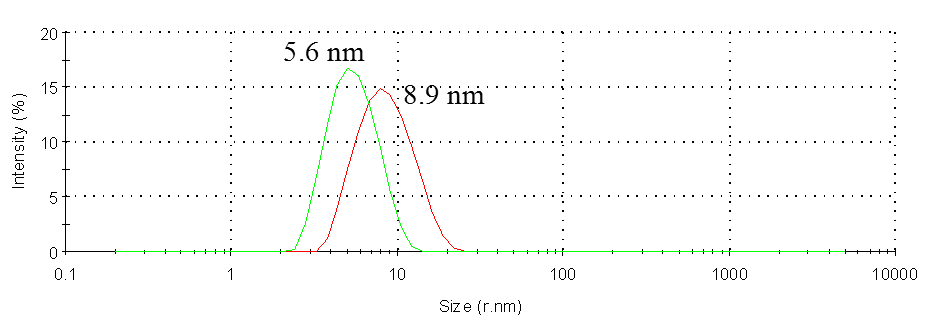


**Supplementary Figure S1.** Dynamic light scattering analysis of fusion proteins. (a) PTDH-PCNA1_G108C_-PdR (red line) and PCNA1_G108C_-PdR (green line), (b) PTDH-PCNA2_L171C_-PdX (red line) and PCNA2_L171C_-PdX (green line) and (c) PTDH-PCNA3_R112C/T180C_-P450cam (red line) and PCNA3_R112C/T180C_-P450cam (green line). Fusion of PTDH to PCNA1_G108C_-PdR, PCNA2_L171C_-PdX and PCNA3_R112C/T180C_-P450cam increased the diameter by two times indicating the homodimeric states of fusion proteins as a result of PTDH homodimerization.

**
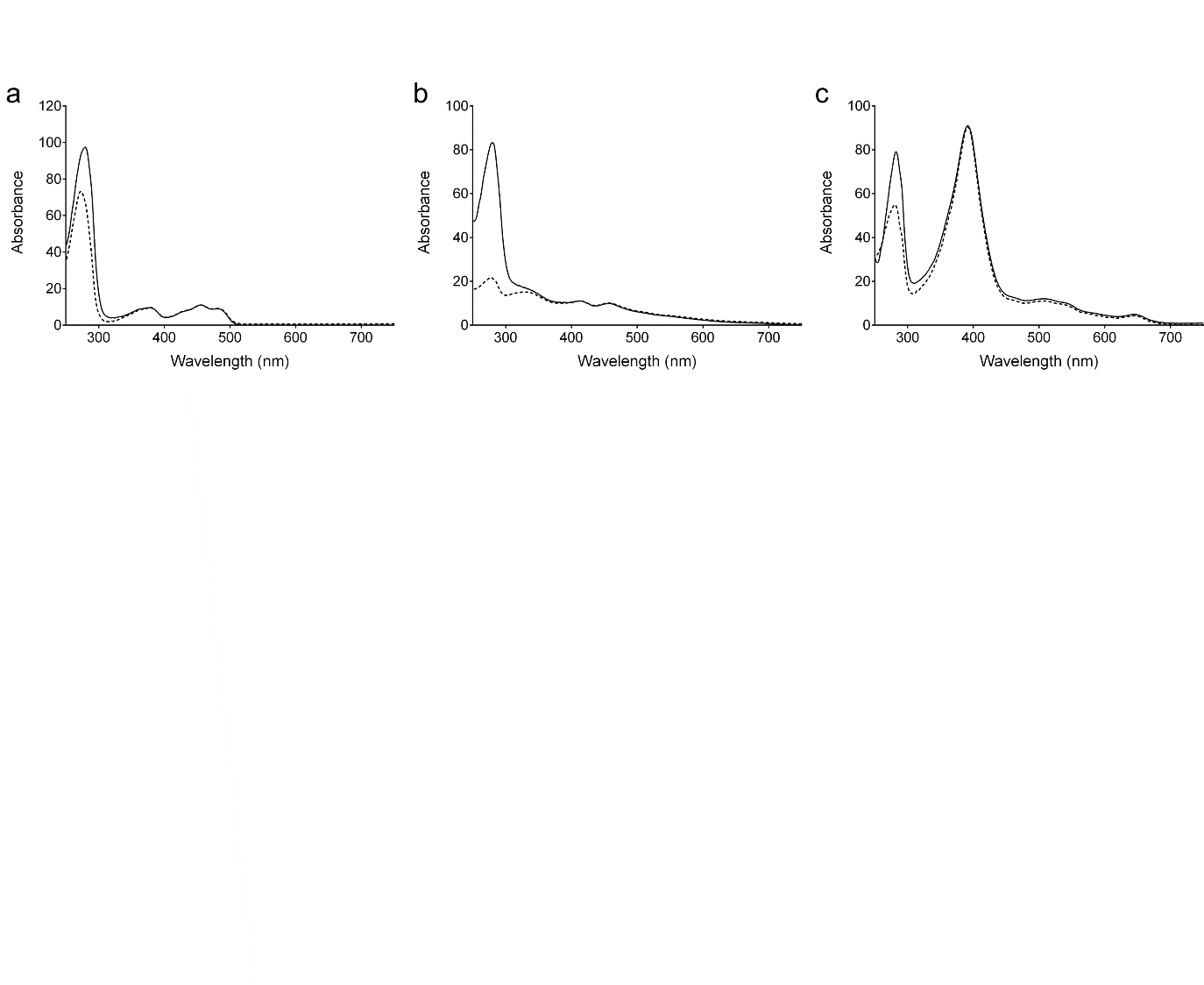
Supplementary Figure S2.** UV-vis spectra of fusion proteins. (a) PTDH-PCNA1_G108C_-PdR (solid line) and wild-type PdR (broken line), (b) PTDH-PCNA2_L171C_-PdX (solid line) and the C73S/C85S mutant of PdX and (broken line) and (c) PTDH-PCNA3_R112C/T180C_-P450cam (solid line) and wild-type P450cam (broken line) in the presence of d-camphor.


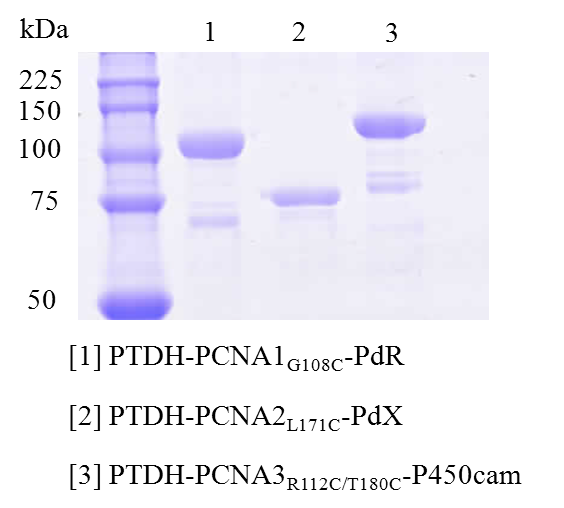


**Supplementary Figure S3.** SDS-PAGE analysis of the triple fusion proteins, PTDH-PCNA1_G108C_-PdR (lane 1), PTDH-PCNA2_L171C_-PdX (lane 2) and PTDH-PCNA3_R112C/T180C_-P450cam (lane 3).


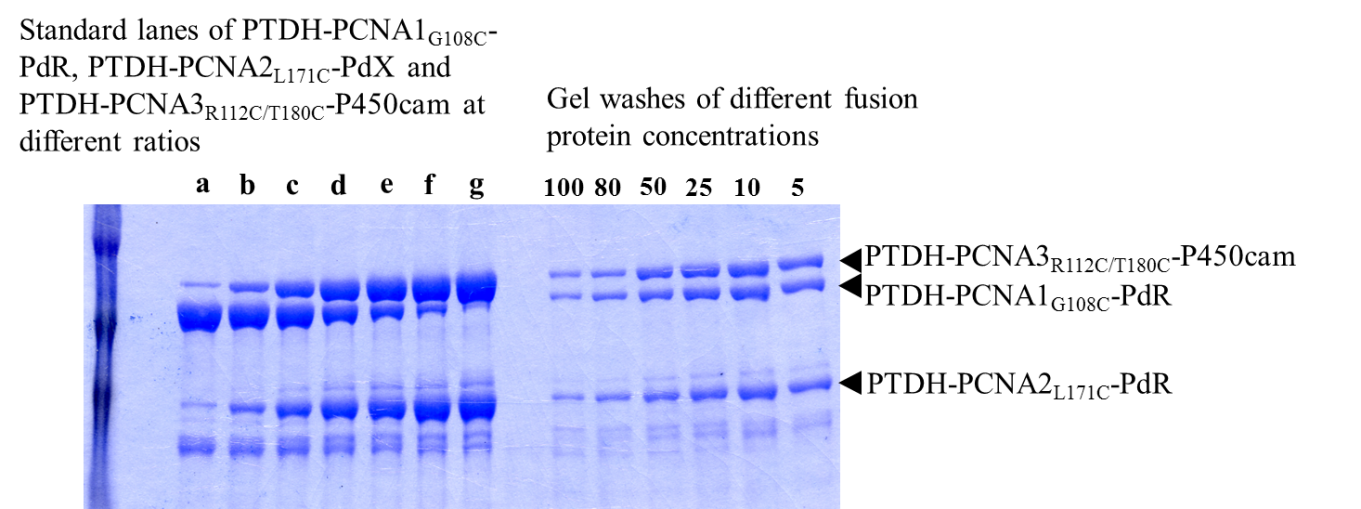


**Supplementary Figure S4.** SDS-PAGE analysis of the combined mixture of the supernatant and three washes.


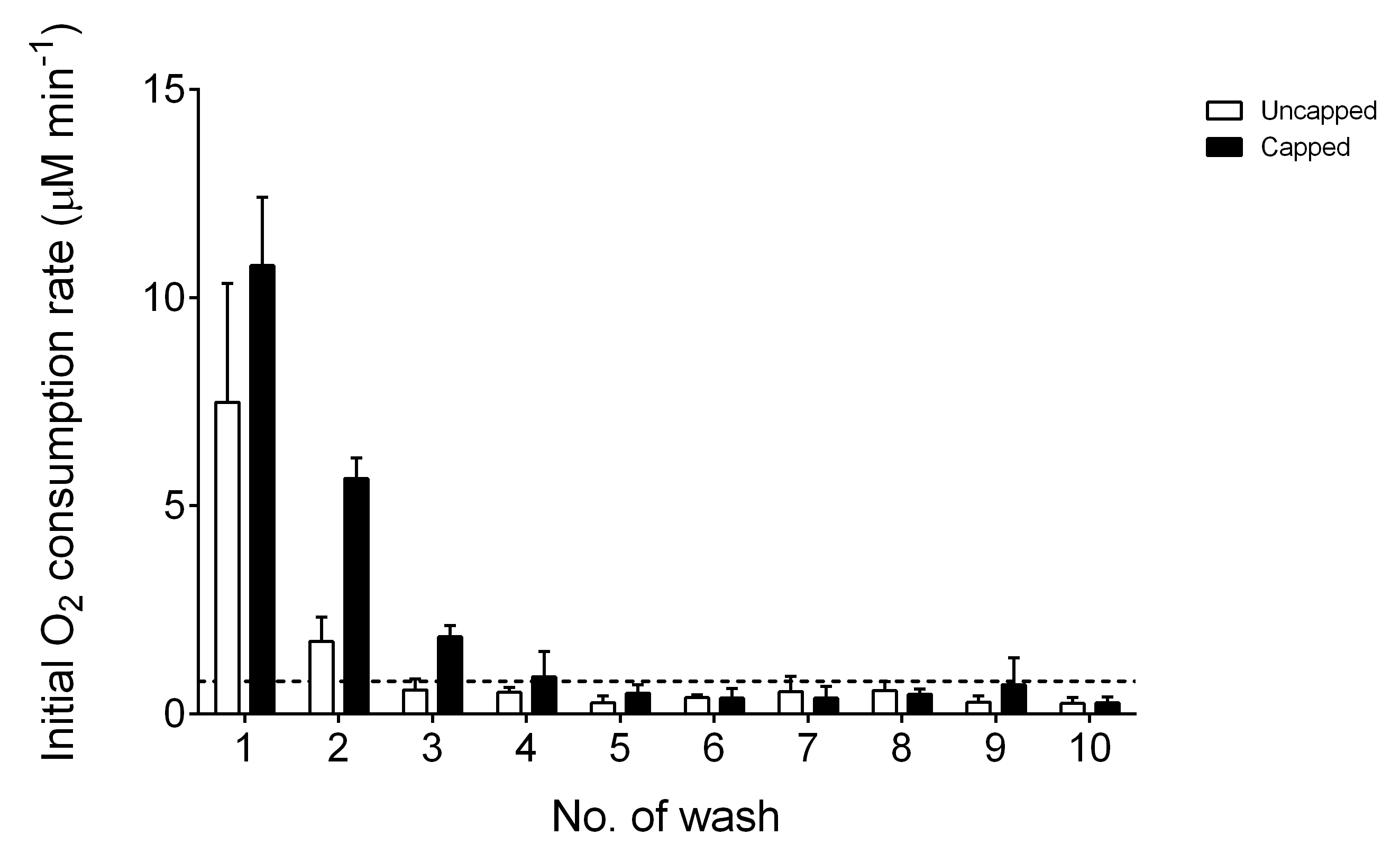


**Supplementary Figure S5.** Oxygen consumption rates of wash solutions of the capped gel (open bar) and the uncapped gel (closed bar). Broken lines indicate background oxygen consumption rates. Error bars represent the standard deviations of three replicates.

**a
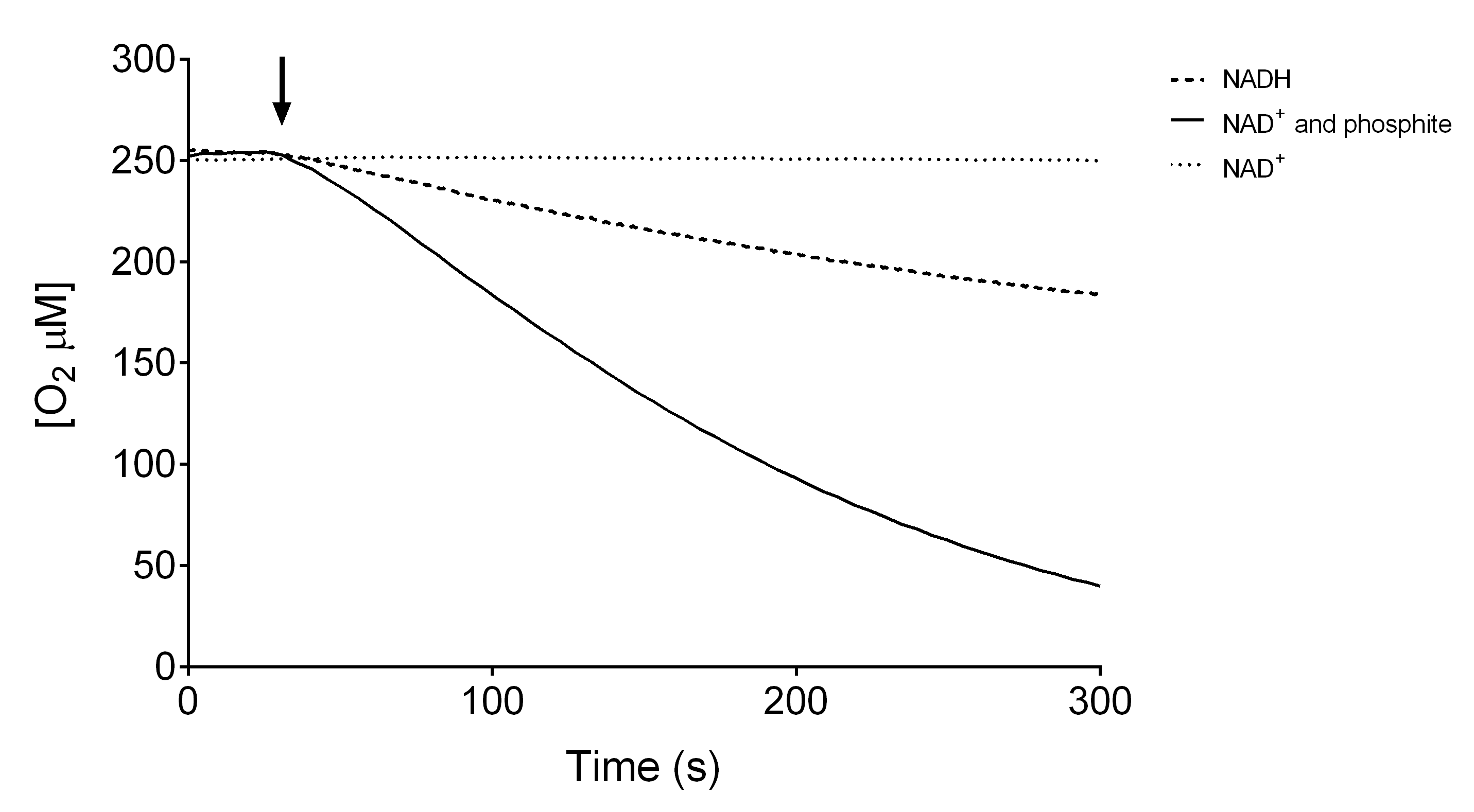
**

**b
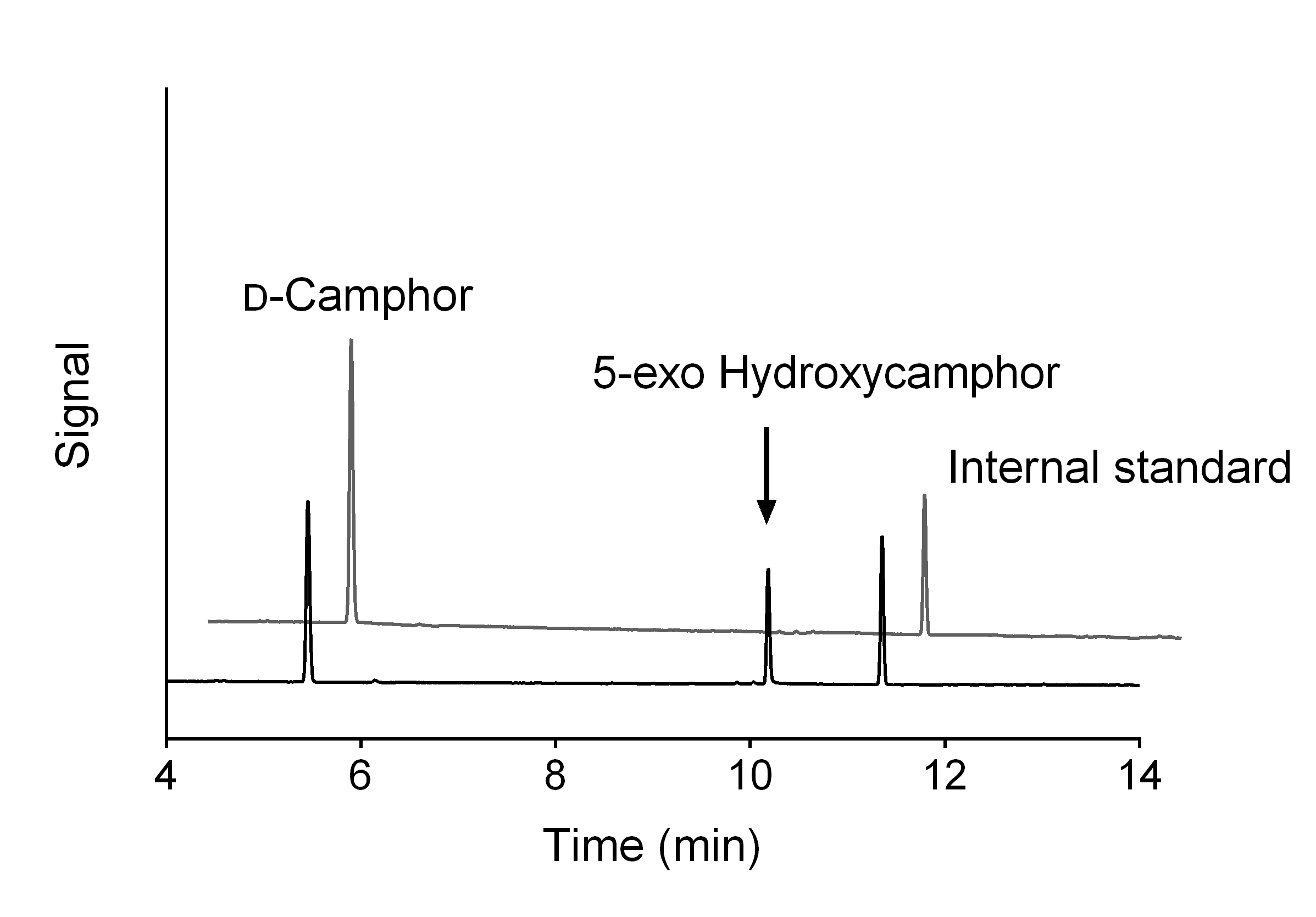
**

**Supplementary Figure S6. (a)** Oxygen consumption of the uncapped gel in the presence of different cofactors and substrates. Oxygen concentration was monitored in the presence of 100 µM NAD^+^ and 10 mM phosphite (solid line), 100 µM NADH (dashed line), or 100 µM NAD^+^ (dotted line) at 25°C. An arrow indicates the addition of cofactor. (b) Gas chromatography profiles of a reaction mixture containing 1 mM d-camphor, 0.5 mM NADH and the capped gel prepared from 30 µL of 80 µM protein mixture (black line), and a control reaction mixture (grey line). Presence of product peak, hydroxycamphor was distinctively detected at retention time of 10.2 min.

**a**
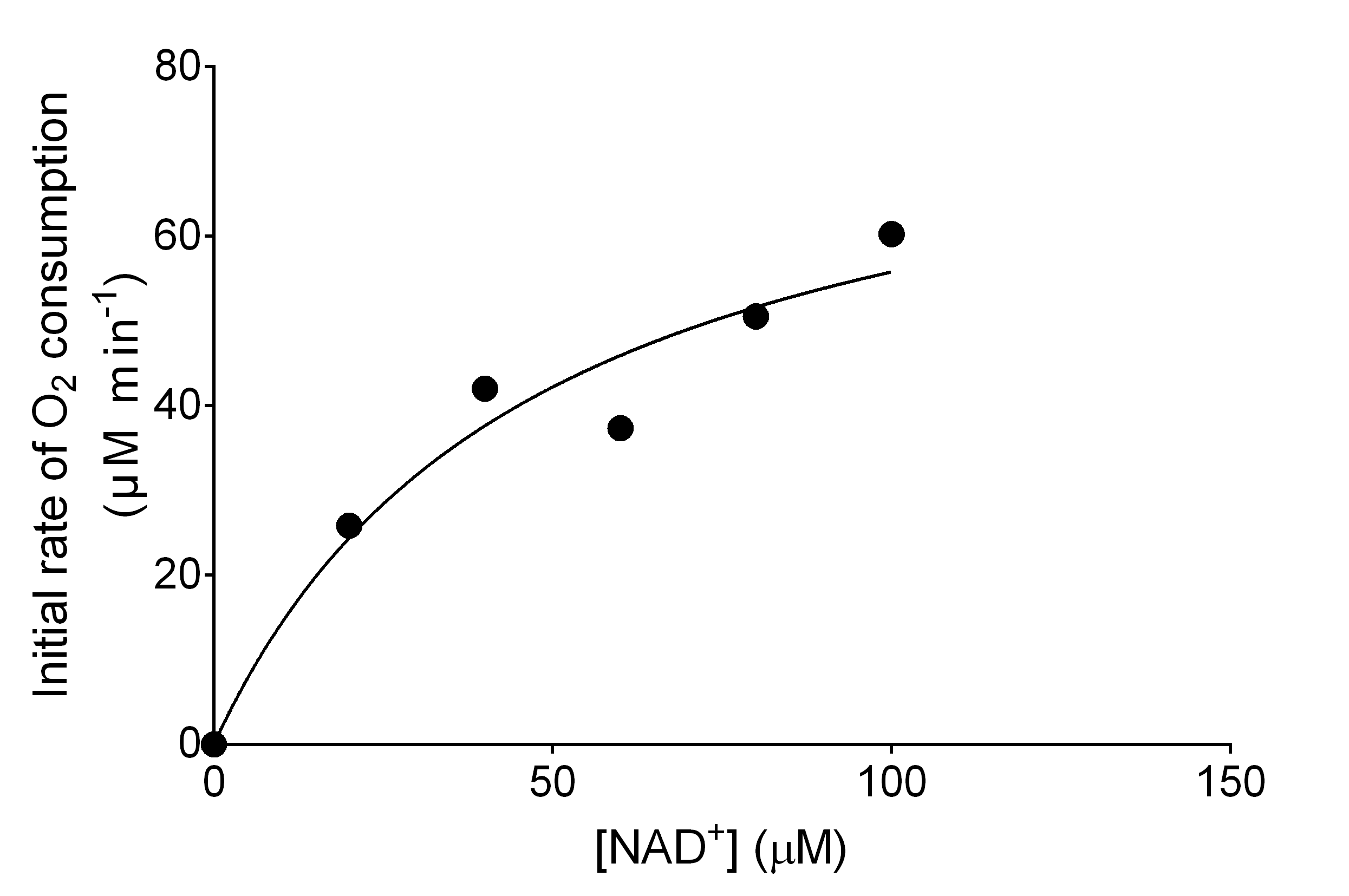


**b**
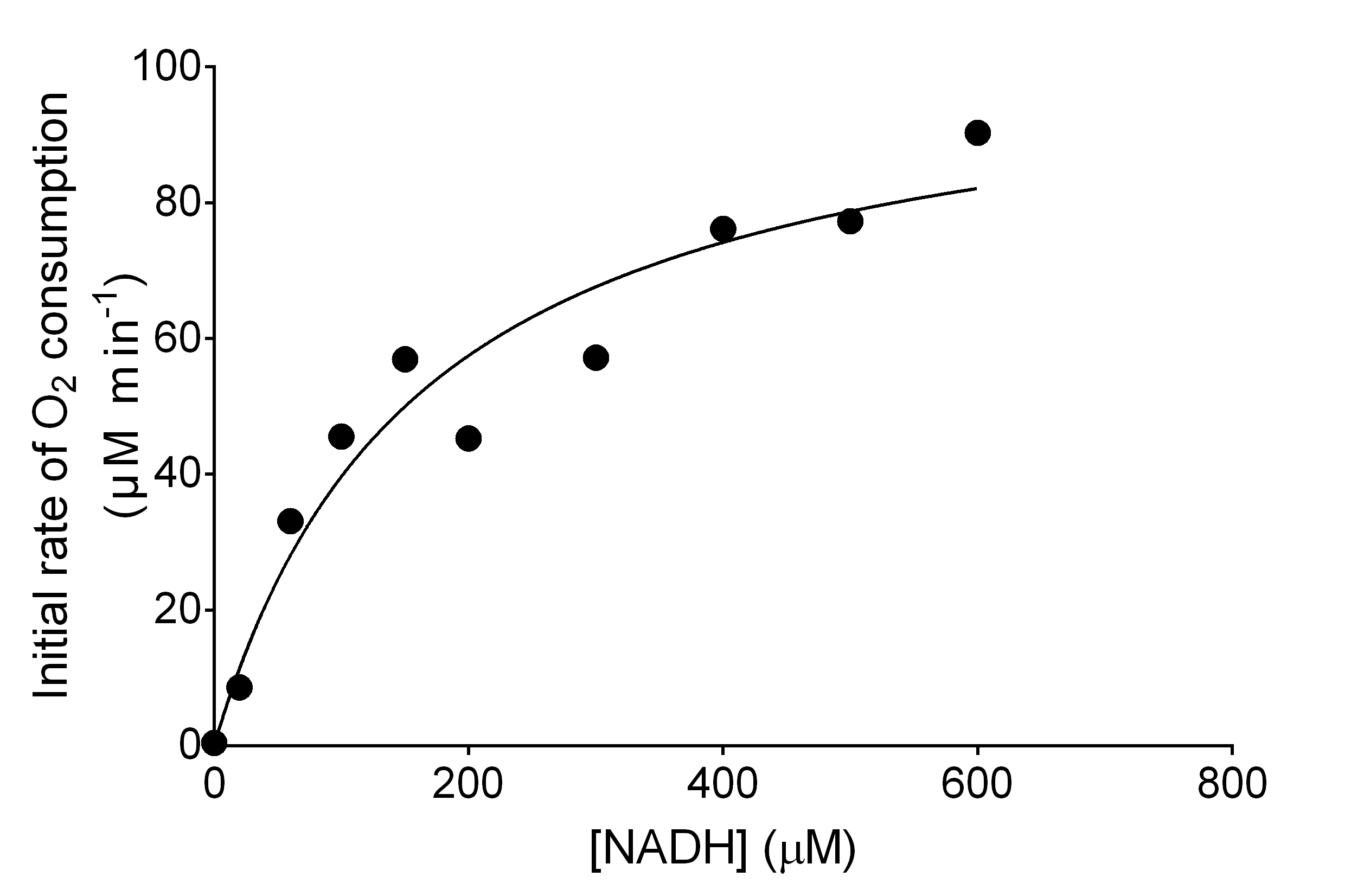


**Supplementary Figure S7.** Michaelis-Menten plots of capped gel in the presence of different cofactors. Kinetic studies were performed in (a) 100 µM NAD^+^ and 10 mM phosphite for cofactor regeneration and (b) 100 µM NADH without cofactor regeneration.


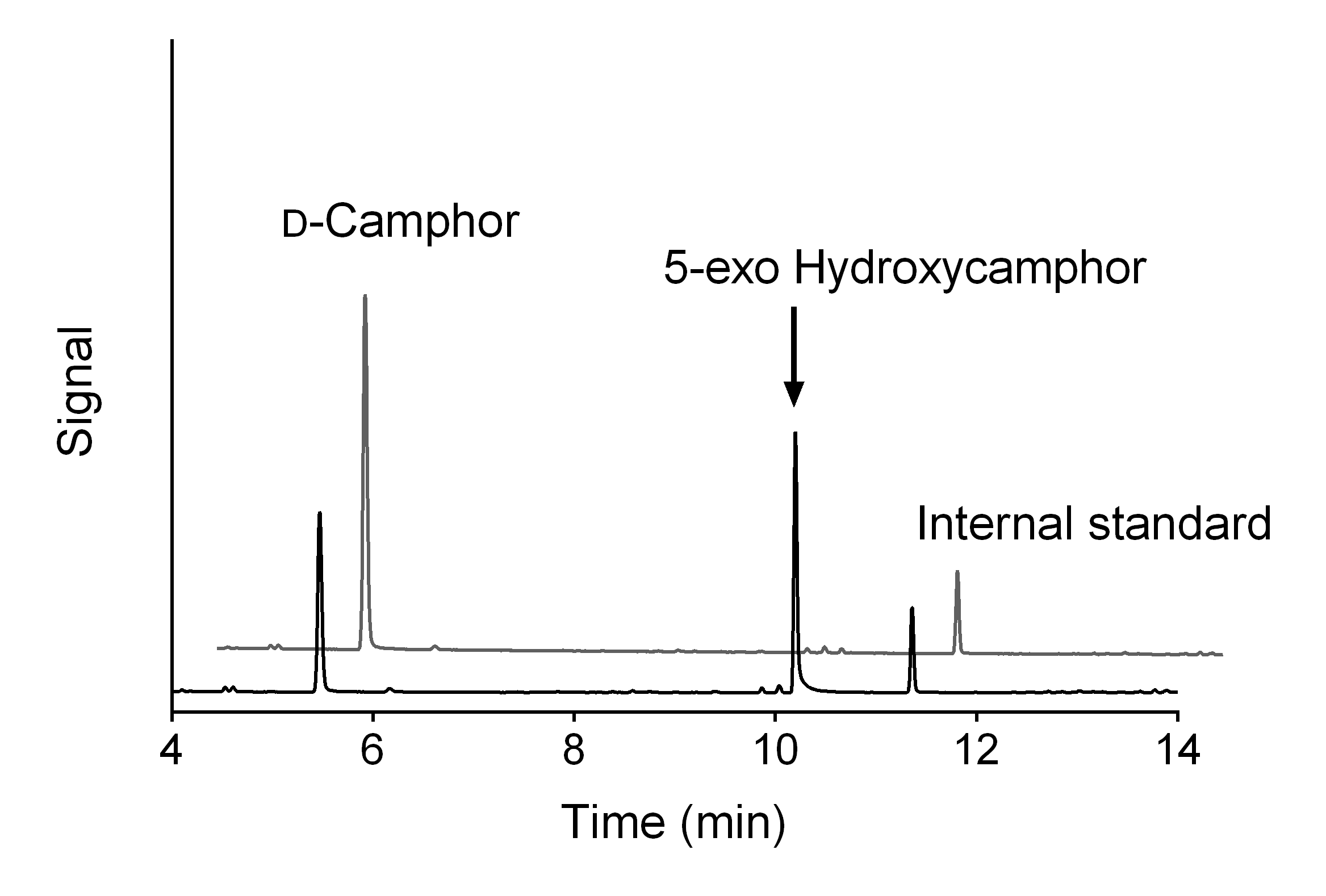


**Supplementary Figure S8.** Gas chromatography profiles of a reaction mixture containing 3 mM d-camphor, 100 µM NAD^+^, 5 mM phosphite and the capped gel prepared from 30 µL of 80 µM protein mixture (black line), and a control reaction mixture (grey line) in the first cycle of reusability experiment. Presence of product peak, hydroxycamphor was distinctively detected at retention time of 10.2 min.

**Supplementary Equations**

The reaction scheme for the overall reaction that contributes to oxygen consumption in the regeneration system of free enzymes is as follows:


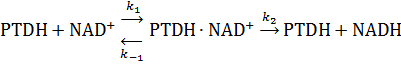


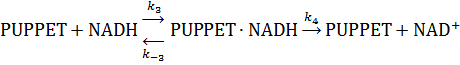


The rate of change for each species can be represented by the following equations:


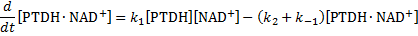


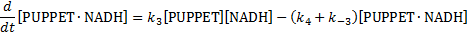


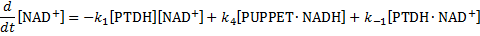


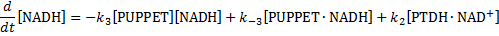


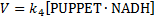


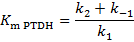


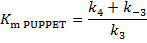


Total concentrations of PUPPET, PTDH and cofactor can be represented by the following equations:


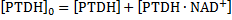


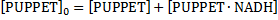


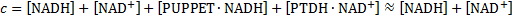


Therefore,


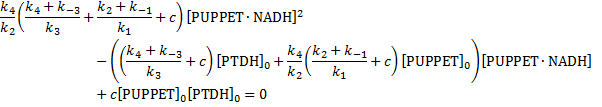


When *c* = ∞,


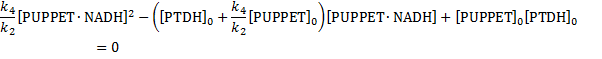


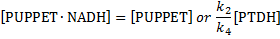


*V* = *V*_max_

Therefore,


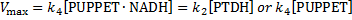


When *c* = *K*_m_,


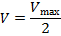


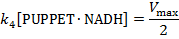


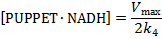


Therefore,


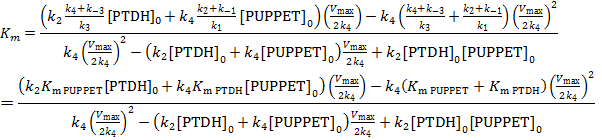


**Supplementary References**

[S1] Hirakawa, H., Kakitani, A. & Nagamune, T. Introduction of selective intersubunit disulfide bonds into self-assembly protein scaffold to enhance an artificial multienzyme complex’s activity. *Biotechnol. Bioeng.* **110,** 1858–64 (2013).

[S2] Watanabe, H., Hirakawa, H. & Nagamune, T. Phosphite-driven Self-sufficient Cytochrome P450. *ChemCatChem* **5,** 3835–3840 (2013).

[S3] Hirakawa, H. & Nagamune, T. Molecular assembly of P450 with ferredoxin and ferredoxin reductase by fusion to PCNA. *Chembiochem* **11,** 1517–20 (2010).

[S4] Sevrioukova I. F. *et al.* Crystal structure of putidaredoxin, the [2Fe-2S] component of the P450cam monooxygenase system from *Pseudomonas putida*. *J. Mol. Biol.* **333**, 377–392 (2003).
